# Selective Lysine Ubiquitination Using Activated Phenol Esters

**DOI:** 10.1002/cbic.202500266

**Published:** 2025-07-30

**Authors:** Halana C. Vlaming, Yara Huppelschoten, Rayman T. N. Tjokrodirijo, Peter A. van Veelen, Francesca D’Amico, Kim B. Jensen, Jens Buchardt, Thomas E. Nielsen, Bhavesh Premdjee, Gerbrand J. van der Heden van Noort

**Affiliations:** ^1^ Dept. Cell and Chemical Biology Leiden University Medical Centre 2333 ZC Leiden The Netherlands; ^2^ Global Research Technologies Novo Nordisk A/S Novo Nordisk Research Park DK‐2760 Måløv Denmark; ^3^ Centre for Proteomics and Metabolomics Leiden University Medical Centre 2300 RC Leiden The Netherlands; ^4^ CMC API Development Novo Nordisk A/S Novo Nordisk Park DK‐2880 Bagsvaerd Denmark

**Keywords:** activated ester, lysine, peptide, ubiquitination

## Abstract

Ubiquitination of target proteins is an essential post‐translational modification influencing a wide variety of cellular processes. Herein, the use of a novel water‐soluble acylation reagent based on the 2,4‐dichloro‐6‐sulfonic acid phenol ester of ubiquitin is described for efficient and selective ubiquitin modification of peptides. Under alkaline conditions, this reagent is swift and regioselective toward lysine acylation, while at neutral pH it shows loss of regioselectivity and is able to acylate both lysine and N‐terminal modification at reduced speeds. As proof of concept, a model peptide is utilized to demonstrate this strategy, proving to be successful. Then the ubiquitination of a synthetic protein called Fau gene encoded Ubiquitin‐like protein (FUBI) is performed under alkaline conditions followed by tandem MS analysis, proving that the selective lysine ubiquitination works to prepare protein–protein conjugates.

## Graphical abstract

Ubiquitination of both peptides and proteins using a novel water‐soluble acylation reagent, based on a 2,4‐dichloro‐6‐sulfonic acid phenol ester, is presented. At alkaline pH, fast lysine ubiquitination is observed on model peptide FLYRANK, and its regioselectivity is confirmed using controls. The same selective lysine ubiquitination is shown on the Fau gene encoded Ubiquitin‐like protein (FUBI) protein, which is confirmed using liquid chromatography tandem mass‐spectrometry analysis/mass spectrometry analysis.

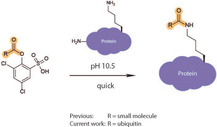

## 1 Introduction

Post‐translational modification of proteins with ubiquitin (Ub) is essential in cellular signaling and plays a key role in processes such as cell division, cell differentiation, and cell survival in all eukaryotes.^[^ ^1^ ^]^ The first step in Ub‐dependent signaling is the covalent attachment of Ub to substrate proteins catalyzed by a triad of enzymes; E1‐activating enzyme, E2‐conjugating enzyme, and E3 ligase enzyme^[^ ^2^, ^3^, ^4^ ^]^ that lead to the transfer of Ub to (most commonly) a lysine ε‐amine in the target protein.^[^ ^3^ ^]^ Substrates can be decorated with a single or multiple Ub monomers or with homotypic poly‐Ub chains consisting of one specific linkage type, wherein Ub's are interconnected via the M1, K6, K11, K27, K29, K33, K48, or K63 residues of Ub. Research on the effects of protein ubiquitination, as well as the mechanisms and selectivities of (de)ubiquitinating enzymes, has benefited from well‐defined materials such as poly‐Ub‐chains, Ub‐based reagents and probes, and ubiquitinated peptides and proteins.^[^ ^5^ ^]^ Significant biochemical effort has been put in the enzymatic synthesis of such materials, mostly relying on specific E2/E3 enzyme combinations.^[^ ^6^ ^]^ Chemical protein synthesis has proven to be an effective alternative to afford Ub tools with high homogeneity in workable quantities.^[^ ^7^, ^8^, ^9^, ^10^, ^11^, ^12^ ^]^ Formation of ubiquitinated peptides was first accomplished by facilitating a native chemical ligation (NCL) approach, using photolabile thiol‐containing auxiliary groups introduced by Chatterjee et al.^[^ ^13^ ^]^ This method has been widely expanded by using various improved thiolysines like 4‐mercapto‐L‐lysine^[^ ^14^, ^15^, ^16^ ^]^ or using other building blocks,^[^ ^17^ ^]^ like oxime‐linkages by functionalizing the C‐terminus of ubiquitin with an aldehyde followed by ligation with an aminoxy‐modified peptide.^[^ ^18^ ^]^ Tang et al. used a preconstructed isopeptide‐linked 76‐mer (isoUb) to synthesize atypical Ub chains together with classical Cys‐based NCL, or using an isopeptide‐linked ubiquitin isomer to make polyubiquitin chains.^[^ ^19^, ^20^ ^]^ The described methods show product formation, however, not without a few disadvantages. For example, thiolysines are not commercially available, hence long syntheses are needed to install the *S‐tert*‐butyl disulfide group on the peptide.^[^ ^14^ ^]^ Additionally, native chemical ligation requires activated thioester reactants that suffer from hydrolysis and desulphurization steps after the product is formed, resulting in loss of product and therefore having a low yield. Another way to create polypeptides is via lysine acylation. Lysine acylation of polypeptides in aqueous media has been reported over the last decade; however, there are still challenges regarding selectivity and efficiency.^[^ ^21^, ^22^, ^23^ ^]^ For example, when using highly activated esters, such as N‐hydroxy succinimide (NHS) and *p*‐nitrophenol (*p*NP) esters, there is the possibility of transesterification with serine, threonine, and tyrosine residues in biomolecules.^[^ ^24^, ^25^ ^]^

**Figure 1:**
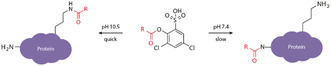
pH dependent selective lysine acylation at pH 10.5 (left arrow) and N‐terminal acylation at pH 7.4 (right arrow) in aqueous conditions. In previous work, R = small cargo. In the current work, R = synthetic Ub protein.

Such side reactions are not observed when using the acylating reagent 2,4‐dichloro‐6‐sulfonic acid phenol ester described by Jensen et al.^[^ ^26^, ^27^ ^]^ In their study, they attach the acylation reagent to small molecules such as small PEG‐linkers or aromatic moieties, followed by ligation of a peptide. They screened a panel of reagents based on the 2,4‐dichloro‐6‐sulfonic acid phenol ester core with various linker structures, and found that they are stable toward hydrolysis under alkaline aqueous conditions. At high pH, the reagents tend to be regioselective for lysine acylation while at neutral pH they show high preference for N‐terminal modification (**Figure** **1**). To achieve selectivity if multiple lysine residues are available in either the acyl‐donor or acceptor, orthogonal protection or mutation strategies need to be taken into account. Earlier reports on employing such protection group strategies in Ub chain synthesis show the applicability of such methods.^[^ ^28^, ^29^, ^30^ ^]^ We envisioned that such 2,4‐dichloro‐6‐sulfonic acid phenol esters might be suitable for the synthesis of ubiquitinated peptides. This method would eliminate the need for additional desulphurization and therefore preserve yield. To affect such an approach, we report on the synthesis of activated phenol ester ubiquitin proteins and subsequent investigation of their lysine versus N‐terminal acylating properties on model peptides.

We synthesized a mutant ubiquitin ester activated with the 2,4‐dichloro‐6‐sulfonic acid phenol group, and showed the selective acylation of a lysine in a model peptide at alkaline pH. We moved on toward a larger protein FUBI, where we observed the same result, which was confirmed using tandem mass spectrometry experiments.

## 2 Results and Discussion

**Scheme 1:**
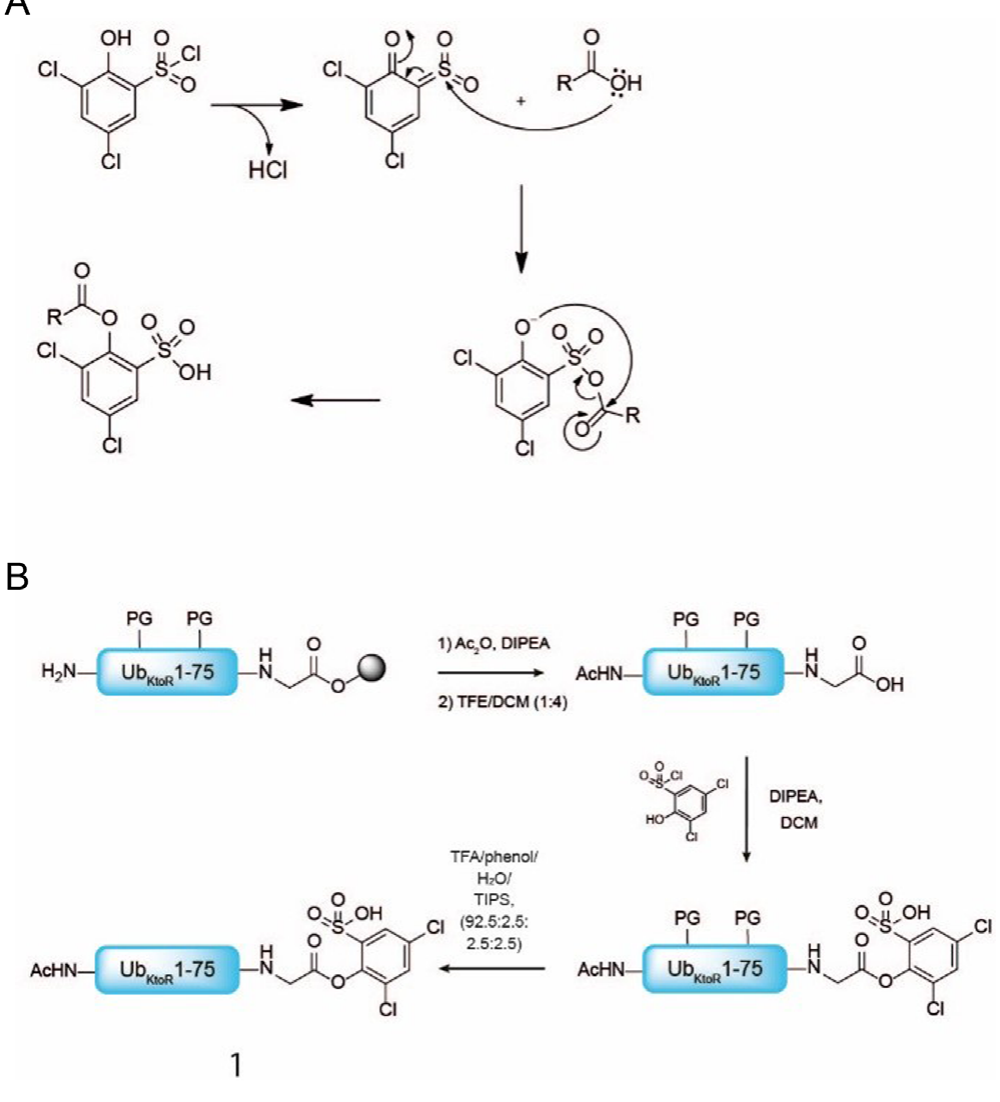
A) Rearrangement of the hydroxybenzenesulfonyl chlorides forming a sulfoquinone, followed by the nucleophilic attack of a carboxylic acid. B) Synthetic route toward Ub_KoR_ ester **1**.

The activated esters used in this study are based on a low‐epimerizing peptide coupling reagent introduced by Cabaret et al.^[^ ^31^ ^]^ where hydroxybenzenesulfonyl chlorides are transformed to sulfoquinones followed by a nucleophilic attack of the carboxylic acid (**Scheme** **1**A). This leads to an intramolecular transfer reaction resulting in an activated ester. Jensen et al succeeded in acylation of peptides with small molecular cargo, including amino acids, lipids, or biotin groups, using these hydroxybenzenesulfonyl chlorides as coupling reagent. Therefore, we aimed to investigate whether this same activated ester strategy could be applied in the activation and selective lysine acylation with larger cargo such as proteins like ubiquitin. As the difference of hydrolytic stability of the activated ester between different donors can vary, we wondered if a ubiquitin‐ester would be a suitable acylation reagent. Ubiquitin, however, can be a complex acylating molecule since either of the seven lysines or N‐terminus in Ub itself can act as a donor site. Hence, we mutated all lysines to arginines (peptide **1**) and blocked the N‐terminus with an acyl group to prevent self‐acylation of Ub either intermolecular or intramolecular.

The Ub_KtoR_ activated ester **1** was prepared by synthesizing Ub_1‐76_ using Fmoc solid‐phase peptide synthesis (SPPS) on 2‐chlorotrityl chloride resin followed by acetylating the N‐terminus using acetic anhydride and N,N‐diisopropylethylamine (DIPEA). Afterward, the resin was cleaved using mild acidic conditions (2,2,2‐trifluoroethanol (TFE)/dichloromethane (DCM)) to liberate the C‐terminal carboxylic acid while leaving all side chain protecting groups in place. After release from the resin, the protected peptide was dissolved in DCM and 1.1 equiv. of 2,4‐dichlorophenol‐6‐sulfonyl chloride was added together with 4 equiv. of DIPEA, resulting in a bright yellow solution at pH 8. After 30 min, the reaction mixture turned pale yellow, indicating that the activation was complete, as was also confirmed using liquid chromatography–MS (LC–MS) analysis. Acid‐promoted cleavage of all protecting groups followed by RP‐high‐performance liquid chromatography purification resulted in activated Ub **1** with an overall isolated yield of 12.6% based on initial loading of the resin (Scheme 1B).

**Figure 2:**
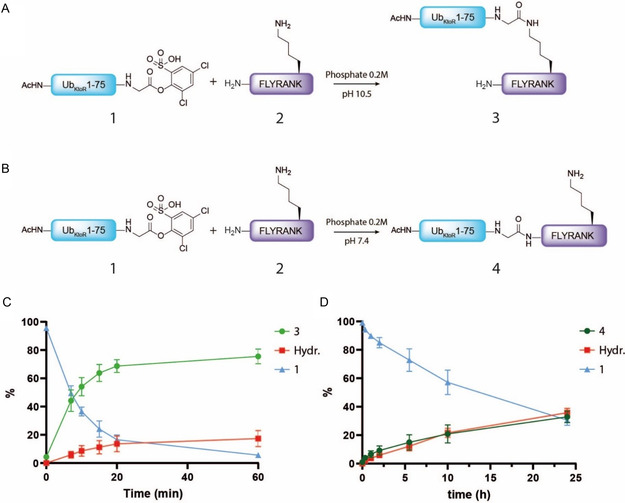
A) Reaction scheme of acylation of FLYRANK model peptide **2** (5 eq) with Ub_KtoR_ ester **1** (1 eq) at pH10.5. at a final concentration of 0.75 mM. B) Reaction scheme of acylation of FLYRANK model peptide **2** (5 eq) with Ub_KtoR_ ester **1** (1 eq) at pH 7.4. at a final concentration of 0.75 mM. C) Formation of Ub_KtoR_‐FLYRANK **3** (light green line), Ub‐ester **1** hydrolysis (red line), and the remaining starting material **1** (blue line) as relative percentage determined using LC‐MS analysis. The reaction at pH 10.5 is monitored over time. D) Formation of Ub_KtoR_‐FLYRANK **4** (dark green line), Ub‐ester **1** hydrolysis (red line) and remaining starting material **1** (blue line) as relative percentage. The reaction at pH 7.4 is monitored using HRMS over time. All measurements were conducted in triplicate and plotted as mean value with indicated standard deviation.

Our studies on the selective lysine ubiquitination of peptides commenced with the acylation of test peptide H‐FLYRANK (peptide **2,** Figure S2, Supporting Information) by activated Ub **1** (**Figure** **2**A). Unlike model peptide H‐GLYRANK, which was chosen before by Mikkelsen et al,^[^ ^26^ ^]^ FLYRANK has an N‐terminal phenylalanine, making it easy to follow on ultra high‐performance liquid chromatography ‐ mass spectrometry by monitoring the UV absorption. Peptide **2** was dissolved in freshly prepared phosphate buffer (0.2 M Na_2_HPO_4_, 0.2 M NaH_2_PO_4_) at pH 10.5. Activated Ub ester **1** was dissolved in dimethylsulfoxide and added slowly to peptide **2** in solution. The resulting suspension was shaken at 25 °C at 350 rpm, and reaction progress was monitored by high‐resolution liquid chromatography‐MS (HRMS) over time (Figure 2C).

We observed that in the first 10 min, the product formation rate is significant and after 10 min, conversion of the Ub_KtoR_‐FLYRANK conjugate is >50%. After 20 min, the complex increased to a conversion of 64%, and after an hour, to a total conversion of 76%. These results are also in line with the measured amount of remaining activated Ub (**1**) starting material, which drops equally fast as the increase of the product, resulting in a few percent remaining after 1 h. Because the reaction mixture is alkaline, the starting material also hydrolyzes over time. The rate of formation of hydrolysis product, however, is slowly increasing to 17% over 60 min. Overall, these results clearly show that product formation is much more efficient than hydrolysis in the first 20 min.

Compared to native chemical ligation reactions, which often require overnight reaction times and result in high amounts of hydrolyzed thioesters, the short reaction time and minimum hydrolysis observed in this reaction are very appealing.

The same reaction was performed at the lower pH 7.4 (Figure 2B). As N‐terminal acylation is reported to take substantially longer than lysine acylation at pH 10.5, the reaction was measured over a period of 24 h using HRMS (Figure 2D). The conversion to product **4** gradually increases and in 24 h results in a total conversion of 33%. The rate of hydrolysis goes almost hand in hand with the rate of formation of product **4**, while the amount of starting material **1** likewise decreases to a final value of 31%. These results indicate that even though product formation is not able to exceed the rate of hydrolysis of the starting material, the activated ester is surprisingly stable enough not to be completely hydrolyzed over 24 h. This makes this reagent suitable even for challenging conversions that need extended periods of time.

**Figure 3:**
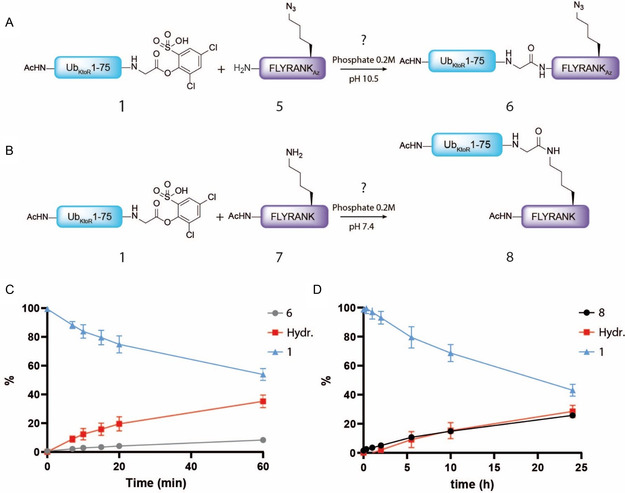
A) Reaction scheme of FLYRANK_Az_ **5** (5 eq) acylation with active ester **1** (1 eq) at pH 10.5 at a final concentration of 0.75 mM as a control for regioselectivity. B) Reaction scheme of Ac‐FLYRANK **7** (5 eq) acylation with active ester **1** (1 eq) at pH 7.4 at a final concentration of 0.75 mM as a control for regioselectivity. C) Formation of Ub_KtoR_‐FLYRANK_Az_ **6** (gray line), Ub‐ester **1** hydrolysis (red line), and remaining starting material **1** (blue line) as relative percentage determined using LC‐MS analysis. The reaction at pH 10.5. is monitored using HRMS over time D) Formation of Ub_KtoR_‐Ac‐FLYRANK **8** (black line), Ub‐ester **1** hydrolysis (red line), and remaining starting material **1** (blue line) as relative percentage. The reaction at pH 7.4 is monitored using HRMS over time. All measurements were conducted in triplicate and plotted as mean value with indicated standard deviation.

As mass spectrometric analysis of these reactions did not report on the regioselectivity of N‐terminal versus lysine acylation, we set out to investigate if the activated Ub **1** is truly attached to the lysine at pH 10.5 or to the N‐terminus at pH 7.4. Therefore, two control reactions were executed on peptide (**5)**, where an azido‐lysine (K_Az_) was incorporated into the H‐FLYRANK sequence replacing the regular lysine, precluding this site from acylation by activated Ub ester **1** when performing the reaction on pH 10.5 (**Figure** **3**A). Additionally, an N‐terminally acetylated peptide (**7**) was tested, where the N‐terminus is blocked and unavailable for further ubiquitination at pH 7.4 (Figure 3B). At pH 10.5, where we would expect lysine acylation to be preferential, the progress of the N‐terminal acylation of the H‐FLYRANK_Az_ **5** is monitored. Under these conditions, the N‐terminal ubiquitination leading to **6** only reaches a minimal conversion of <10% after 1 h of shaking (Figure 3C). The hydrolysis of the active Ub ester under these conditions increased significantly when compared to Figure 3C, as no competing free lysine is around and product formation with de N‐terminus proceeds markedly slower. Overall, it can be concluded that the N‐terminal acylation of the reaction is negligible at pH 10.5, which is an extra indication that in Figure 3, the acylation via the lysine is formed. In addition, the significant difference in production rate between products **3** and **6** also supports this claim. The progression of the ubiquitination of the N‐terminally acetylated FLYRANK **7** at pH 7.4 (Figure 3D) shows a similar rate as when compared to regular H‐FLYRANK **4,** reaching 26% in 24 h. Additionally, the difference in product formation between products **8** and **4** (Figure 2D and Figure 3D, respectively) is rather small, making it difficult to distinguish N‐terminal ubiquitination versus lysine ubiquitination under these conditions. Therefore, based on this reaction, we conclude that N‐terminal ubiquitination cannot be steered at a pH of 7.4, unlike is the case for smaller molecules.^[^ ^26^ ^]^

**Figure 4:**
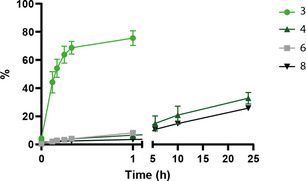
Formation of products **3**, **4**, **6,** and **8** plotted in one figure as relative percentage determined using LC‐MS analysis. Compound **3** shows the Ub_KtoR_‐FLYRANK conjugate formed at pH 10.5 (light green), compound **4** shows the Ub_KtoR_‐FLYRANK conjugate formed at pH 7.4 (dark green). Compound **6** shows the Ub_KtoR_‐FLYRANK_Az_ conjugate formed at pH 10.5 (gray), and compound **8** shows the Ub_KtoR_‐Ac‐FLYRANK conjugate formed at pH 7.4.

When product formation of the above reactions is plotted in one figure in a period of 24 h (**Figure** **4**), it becomes apparent that formation of **3** (lysine ubiquitination at pH 10.5) clearly is the fastest reaction, showing a steep curve, while N‐terminal ubiquitination at pH 7.4 and the control reactions only show marginal product formation. We conclude that quick and effective lysine ubiquitination can be triggered using pH 10.5 within an hour, while at both pH 7.4 and pH 10.5, N‐terminal ubiquitination is only very modest within this short time frame. As protein‐conjugates **4**, **6,** and **8** all show a similar low formation speed far exceeding 1 h to become significant, lysine acylation can hence be selectively steered over N‐terminal acylation.

**Figure 5:**
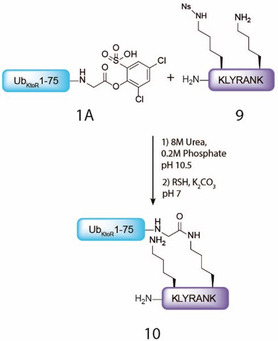
Reaction scheme of acylation of H‐K(Nos)LYRANK model peptide **9** (1.2 eq with activated Ub ester **1A)** 1 eq at a final concentration of 0.5 mM–1 mM.

Next, we continued with the ubiquitination of a model peptide containing two lysine residues of which one is protected with a nosyl protecting group (**9**, Figure S5, Supporting Information). Orthogonal lysine protection would allow site‐selective lysine acylation and open up the way to acylation of more complex peptides/proteins. If the additional lysines are not protected, multiple acylation events are reported to occur,^[^ ^26^, ^27^ ^]^ giving rise to a non‐homogeneous mixture of non‐acylated, mono‐acylated, and di‐acylated products. We envisioned that the nosyl protecting group would be suitable for the protection of the additional lysine position(s), since it can be deprotected using very mild conditions (thiol additive, pH 7). In addition, Fmoc‐Lys(Ns)‐OH is compatible with Fmoc‐SPPS and is easy to synthesize and commercially available. Activated Ub **1 A** and partially protected H‐K^Nos^LYRANK **9** were reacted at pH 10.5. LC‐MS analysis showed full conversion of **1 A** to mono‐ubiquitinated peptide **10** after only 10 min, again with minimal hydrolysis of activated ester **1 A** (**Figure** **5**).

Afterward, removal of the nosyl protecting group of peptide **10** was initiated by adding 100 equiv. 4‐mercaptophenylacetic acid (MPAA) and K_2_CO_3_ at pH 7.^[^ ^32^ ^]^ Overnight incubation resulted in only ≈60% nosyl deprotection, after which the reaction mixture was heated to 37 °C, resulting in complete deprotection after an additional 4 h (Figure S17, Supporting Information). No side products were observed, indicating the compatibility of the nosyl protecting group with our strategy.

**Figure 6:**
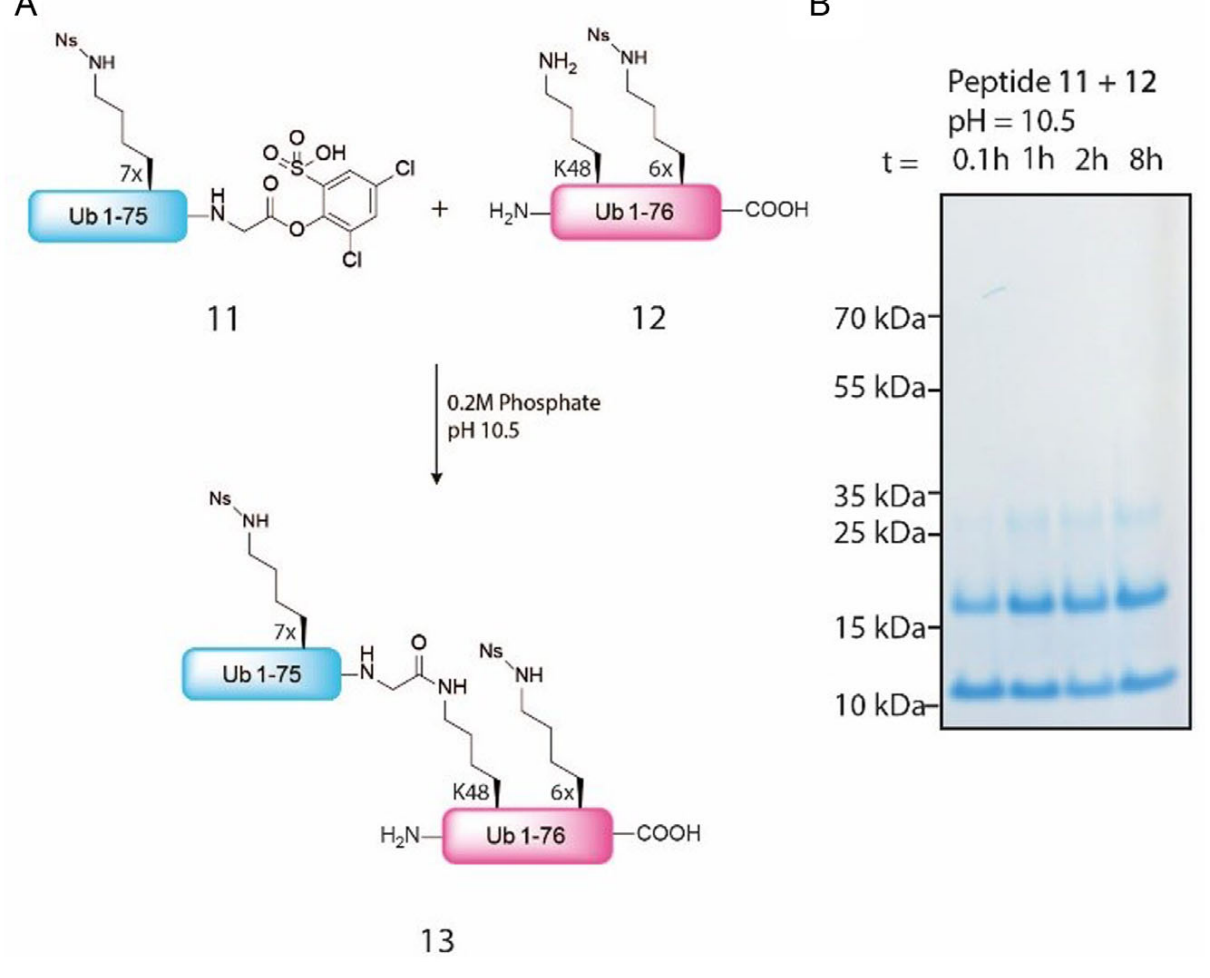
A) Schematic representation of the reaction between synthesized Ub derivatives **11** and **12** at a final concentration of 0.5 mM–1 mM. B) Sodium dodecyl sulfate (SDS)‐Page analysis of acylation reaction of Ub **12** by fully protected activated Ub **11** at pH 10.5 toward Ub dimer **13**.

Encouraged by the results of the selective ubiquitination of model peptides **2** and **9**, we set out to acylate a more complex peptide and decided to attempt to ubiquitinate another ubiquitin protein in order to create diUb chains. Ub, where all lysine residues were nosyl protected, was synthesized and activated (peptide **11**, **Figure** **6**A). In addition, an Ub derivative was synthesized with a single unprotected lysine (Lys48, peptide **12**, Figure 6A), while all other lysine residues were carrying a nosyl group. Hence, the free lysine is the only accessible lysine position for ubiquitination with activated Ub **11**.

Incubation of peptide **11** and **12** at pH 10.5 under buffered conditions led to the swift formation of lysine 48 linked Ub dimer in 10 min as shown by sodium dodecyl sulfate (SDS)‐page analysis (Figure 6B), whereas at pH 7.4 hardly any Ub dimer is formed (Figure S18, Supporting Information) indicating that our method can be used to not only ubiquitinate relatively small peptides but also full proteins given that a suitable protection scheme for lysines is in place. Of note, after prolonged time, a minor amount of Ub trimer can be observed, presumably N‐terminal ubiquitination in agreement with our previous experiments.

**Figure 7:**
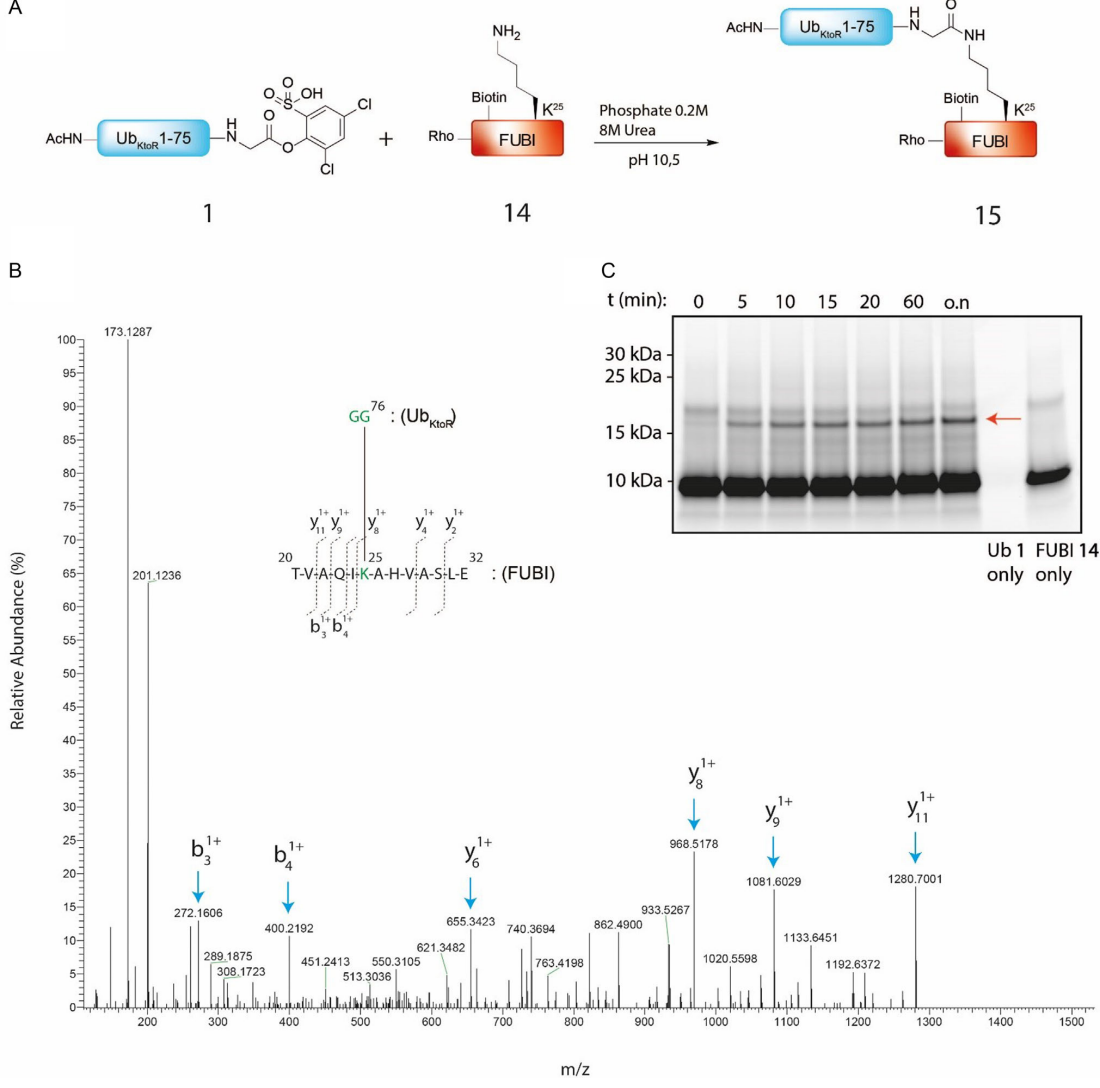
A) Reaction scheme of ubiquitination of FUBI protein **9** (1 eq) with Ub_KtoR_ ester **1** (2 eq at pH 10.5 at a final concentration of 0.31 mM. B) Annotated MS/MS spectrum identifying the K‐GG linkage between the C‐terminus of Ub_KtoR_ and the lysine on position 25 of FUBI. C) Fluorescent scan of SDS‐Page analysis of acylation of FUBI with activated Ub (**1**) followed over the indicated time period (λ_ex/em_ = 473/530 nm).

FUBI, an N‐terminal fusion protein which is synthesized from the FAU gene, owns 36% homology with ubiquitin including the C‐terminal diglycine motif.^[^ ^33^, ^34^ ^]^ Although studies report immunosuppressant roles via conjugation (FUBI‐ylation) via isopeptide bonds with various substrates like BCL‐G^[^ ^35^ ^]^ or T cell antigen receptor‐α,^[^ ^36^ ^]^ its function is largely unexplored. As such, the existence of poly‐FUBI chains or mixed Ub‐FUBI conjugates and enzymes potentially recognizing and reacting with them until now remains under‐explored speculation. Chemical tools prepared for the FUBI protein can therefore be quite instrumental in answering such questions and enlightening yet unknown functions of FUBI. Opposed to ubiquitin, FUBI owns only one lysine residue on position 25,^[^ ^34^ ^]^ which additionally makes this protein extra suitable for our selective ubiquitination at alkaline pH (**Figure** **7**A). The FUBI protein was synthesized using Fmoc SPPS, and the N‐terminus was blocked using a dual rhodamine and biotin modification for visualization using fluorescence scans of SDS‐page gel and pulldown purposes, respectively. FUBI (**14**) was dissolved in phosphate buffer (8M Urea, 0.2 M Na_2_HPO_4_) with denaturation agent urea, to make the K_25_ of FUBI as accessible as possible. After the pH was adjusted to pH 10.5, the solution was stirred for 15 min at 25 °C at 350 rpm. Afterward, Ub **1** was dissolved in DMSO and added slowly to the FUBI solution. The resulting suspension was shaken at 25 °C at 350 rpm and monitored on SDS‐page gel at indicated times (Figure 7C, Figure S19, Supporting Information). Already after 5 min, a band around ≈17 kDa appears, as can be seen in the fluorescence scan of the gel (red arrow). The band increases in thickness at the consecutive timepoints, and the conjugate has the apparent molecular weight of the Ub_KtoR_‐FUBI conjugate (**15**), although overall conversion is modest. To validate that indeed the hybrid FUBI‐Ub conjugate is formed using our selective lysine ubiquitination on K_25_ of FUBI, we digested the formed conjugate with endo‐Glu C and trypsin peptidases and used LC–MS/MS analysis (Figure 7B). The peptide belonging to amino acids 20–32 of the FUBI protein, containing the K‐GG remnant of Ub, was identified. The single‐charged y‐ion series indeed verifies that the K_25_ is the amino acid carrying the GG linkage connecting the two proteins. This data confirms that the Ub_KtoR_‐FUBI complex **15** is formed, demonstrating that the selective lysine ubiquitination at pH 10.5 was also successful for this hybrid Ub **1** chain.

## 3 Conclusion

We herein describe the use of activated phenol esters in selective protein–protein acylation, using pH‐dependent selectivity. Lysine ubiquitination occurs swiftly in an alkaline environment at pH 10.5. We demonstrated this reference in our initial experiments by incubating an activated Ub ester (**1**) with model peptide H‐FLYRANK (**2**) under both alkaline and neutral conditions. Our data shows that lysine acylation at pH 10.5 is fast and selective, and minimum hydrolysis of the activated ester is observed, making this approach an attractive alternative to thiolysine‐based native chemical ligation‐desulfurization methods to prepare ubiquitinated peptides. Additionally, a control reaction using H‐FLYRANK_Az_ assessing the regioselectivity of the reaction showed slow and minimal N‐terminal product formation with a conversion lower than 10% after one hour, further indicating that the lysine is selectively ubiquitinated when using regular H‐FLYRANK. Selective N‐terminal ubiquitination at pH 7.4 provided less concise results, as the difference between production of regular H‐FLYRANK and its control reaction Ac‐FLYRANK is almost nonexistent. Therefore, it is hard to confirm regioselectivity at pH 7.4 based on only this control reaction. These results, however, do show that, as this reaction takes 24 h to complete, the activated Ub ester **1** is able to withstand hydrolysis for an extended period of time.

Moving onto larger proteins, the selective lysine ubiquitination was tested on both orthogonally protected Ub and FUBI, a ubiquitin‐like protein only carrying one lysine at position 25. We did not isolate the final ubiquitylated Ub(l) conjugates to assess yields, but based on these initial results, it becomes apparent that formation of these larger conjugates is less efficient than for the smaller test peptides. SDS‐page gel analysis, however, revealed that already after 5 min a conjugate was formed with the expected mass of the Ub_KtoR_‐FUBI conjugate. This band was analysed using LC‐MS/MS, which confirmed the K‐GG linkage between Ub and the lysine of FUBI. Even though no full conversion was observed, we have shown that selective lysine acylation at pH 10.5 can be used to form protein–protein conjugates, with the reaction being quick and straightforward. Although optimization of an orthogonal protective group strategy for other lysine positions and the accompanying deprotection efficiency as well as blocking of N‐termini to avoid minimal ubiquitination under the used conditions needs more attention, the results shown here highlight the possibilities of this lysine ubiquitination methodology. The stability of the used 2,4‐dichlorophenol‐6‐sulfonyl ester under alkaline conditions proves favorable with respect to thioesters often used in NCL approaches. In combination with selective mutations or protective groups strategies on additional lysine residues, which are not needed in NCL techniques, the present approach might offer a useful alternative to form isopeptide linkages. Further optimization of yields of this ubiquitination protocol could lead to access of Ub dimers and polymers or FUBI chains. The concept can potentially also be further expanded to the synthesis of hybrid chains between Ub and other Ub‐like proteins.

## Conflict of Interest

The authors declare no conflict of interest.

## Supporting information

Supplementary Material

## Data Availability

The data that support the findings of this study are available in the supplementary material of this article.
