# Supplementary material for: Selective Lysine Ubiquitination Using Activated Phenol Esters
Source: Chembiochem. 2025 Jul 30;26(18):e202500266. doi: 10.1002/cbic.202500266 (PMC12447373; doi:10.1002/cbic.202500266)
Supplement: Supplementary file 1 — Supplementary Material [file CBIC-26-e202500266-s001.pdf]

Supporting Information

General procedures

Materials and solvents

Reagents were obtained from Sigma-Aldrich of the highest available grade and used without further purification. Standard Fmoc-protected amino acid derivatives were used and purchased from Gyros Protein Technologies unless mentioned otherwise. Fmoc-Cys(Acm)-OH and resins for SPPS were obtained from Novabiochem (Merck Millipore), Apigenex and PCAS Biomatrix. Pseudoproline dipeptides were obtained from Corden Pharma or Bachem. Iso-acyl dipeptides were obtained from AAPPTec. Solvents for SPPS were obtained from Biosolve. Oxyma Pure® was purchased from Gyros Protein Technologies. HPLC grade acetonitrile was obtained from Merck.

Analytical methods

LC-MS conditions

Method A

LC-MS measurements were performed on a Waters Acquity UPLC H-Class system, using a Waters Acquity BEH 300 Å, C4, 1.7 µm, 2.1 mm x 50 mm (0.4 mL/min), followed by mass detection on a Waters Xevo G2-XS Qtof. Samples were run at 60 °C using 2 mobile phases: A = 0.1 % formic acid in MilliQ water, B = 0.1 % formic acid in acetonitrile (ULC/MS grade) with a gradient of 2 to 100% B over 1 min, 25 to 65 % B over 6 min followed by 65 to 95 % B over 0.5 min maintaining a composition of 5% C throughout. Data processing was performed using Waters MassLynx Mass Spectrometry Software V4.2 (deconvolution with MaxEnt I function). For the first 4.15 min, the flow was diverted from the detector to flush the column with 2% B at 0.6 ml/min to elute most of the buffer components and salt. After 4.15 min, proteins were eluted with a gradient of 2 to 100% B over 1.7 min using a flow rate of 0.6 ml/min. Data processing was performed using Waters MassLynx Mass Spectrometry Software V4.2 (deconvolution with MaxEnt I function), followed by mass detection on a Waters Xevo G2-XS Qtof. Detection range 200-1600 m/z.

Method B

LC-MS measurements were performed on a Waters Acquity UPLC H-Class system, using a Waters Acquity BEH 300 Å, C4, 1.7 µm, 2.1 mm x 50 mm (0.4 mL/min), followed by mass detection on a Waters Xevo G2-XS Qtof. Samples were run at 60 °C using 2 mobile phases: A = 0.1 % formic acid in MilliQ water, B = 0.1 % formic acid in acetonitrile (ULC/MS grade) with a gradient of 2 to 100% B over 2 min using a flow rate of 0.6 ml/min. Data processing was performed using Waters MassLynx Mass Spectrometry Software V4.2 (deconvolution with MaxEnt I function), followed by mass detection on a Waters Xevo G2-XS Qtof. Detection range 200-1600 m/z.

Method C

LC-MS measurements were performed on a Waters Acquity UPLC H-Class system, Waters Xevo G2-XS QTof with a Waters Acquity BEH, C18, 1.7 µm, 2.1 mm x 50 mm (0.4 mL/min). Samples were run at 60 °C using 3 mobile phases: A = 0.1 % formic acid in MilliQ water, B = 0.1 % formic acid in acetonitrile and C = 0.01 % TFA in MilliQ water with a gradient of 5 to 50% B over 6 min, followed by 50 to 95 % B over 0.5 min maintaining a composition of 5% C throughout. Data processing was performed using Waters MassLynx Mass Spectrometry Software V4.2 (deconvolution with MaxEnt I function).

Method D

LC-MS measurements were performed on a Waters Acquity UPLC H Class system, Waters Xevo G2-XS QTof with a Waters Acquity BEH 300 Å, C4, 1.7 µm, 2.1 mm x 50 mm (0.4 mL/min). Samples were run at 60 °C using 3 mobile phases: A = 0.1 % formic acid in MilliQ water, B = 0.1 % formic acid in

acetonitrile and C = 0.01 % TFA in MilliQ water with a gradient of 5 to 25% B over 1 min, 25 to 65 % B over 6 min followed by 65 to 95 % B over 0.5 min maintaining a composition of 5% C throughout. Data processing was performed using Waters MassLynx Mass Spectrometry Software V4.2 (deconvolution with MaxEnt I function).

Solid Phase Peptide Synthesis (SPPS)

Automated Fmoc SPPS

Method 1

SPPS was performed on a Symphony X (Gyros Protein Technologies) automated peptide synthesizer using standard 9-fluorenylmethoxycarbonyl (Fmoc) based SPPS. Fmoc deprotection was achieved with 2 x 10 min. treatment of 20 vol. % piperidine, 0.1 % Oxyma Pure® in DMF. Peptide couplings were performed using DIC/Oxyma. Amino acid/Oxyma solutions (0.3 M/0.3 M in DMF) were added to the resin at 4-6-fold excess together with equal equivalents of DIC (1.5 M in DMF). The coupling time was 2 hours unless specified otherwise. All dipeptide building blocks were coupled for 4 hours. The residual free amino groups after the coupling reaction were capped by the addition of collidine (3.3 equiv., 1.5 M in DMF) and acetic anhydride (10 equiv., 1.0 M in DMF) and were reacted for 20 minutes. After the final Fmoc deprotection the resin was washed with DMF and DCM.

Method 2

SPPS was performed on a SYRO II (Multisyntech, SYRO Robot) automated peptide synthesizer using standard 9-fluorenylmethoxycarbonyl (Fmoc) based SPPS. Fmoc deprotection was achieved with 2 x 2 min. and 1x 5 min treatment of 20 vol. % piperidine in NMP. Peptide couplings were performed using PyBOP/DIPEA. Amino acid solutions (0.34 M in NMP) were added to the resin at 4-fold excess together with an 8-fold excess of DIPEA (1.36 M in NMP) and 4-fold excess of PyBOP (0.34 M in NMP). The coupling time was 2x 25 minutes unless specified otherwise. After the final Fmoc deprotection the resin was washed with NMP and DCM and Et_2O.

Cleavage of protected polypeptides from CTC-resin

Polypeptides were detached from the resin by treatment with TFE/DCM (1:3, v/v), 3x for 1 hour. All filtrates were combined and concentrated under reduced pressure. Followed by co-evaporation of the protected protein by DCM/ACN (1:1).

Release from Chemmatrix resin and global side chain deprotection

Polypeptides were detached from the resin and deprotected by treatment with a TFA cleave mix (TFA/phenol/H_2O/TIPS, 92.5:2.5:2.5:2.5 v/v/v/v/v) for 2-3 hours followed by precipitation in ice cold diethylether and collection by centrifugation. The pellet was resuspended in diethylether before being collected by centrifugation again. The pellet was dissolved in H_2O/CH_3CN/AcOH, 65:25:10, v/v/v and lyophilized before purification.

Purification of peptides

Method 1

Preparative purification was performed on a Waters HPLC equipped with a Waters 2489 UV/Vis detector and Waters fraction collector III using a reversed phase HPLC column as specified in the experimental section. Elution was performed using 2 mobile phases: A = 0.1 % TFA in MilliQ water and B = 0.1% TFA in acetonitrile using a linear gradient. Fractions were collected using a Gilson

fraction collector and relevant fractions were assessed by analytical LC-MS. Fractions containing the pure peptide were pooled and lyophilized.

Method 2

HPLC purifications were performed on a Shimadzu LC-20AT HPLC system equipped with a Shimadzu SPD-20A UV/Vis detector, a Shimadzu FRC-10A fraction collector and a Waters XBridge BEH C18 OBD Prep Column (130Å, 5 µm, 10 x 150 mm) was used. Samples were run with a 30 min gradient detailed in the subsequent method at a flowrate of 6.5 ml/min. Mobile phase: A = 0.05% TFA in MilliQ water and B = 0.05% TFA in acetonitrile. T = 40 °C.

Preparation of peptide fragments

Sequences

Table S1. Amino acid sequence of the peptides synthesized, pseudoprolines are underscored.

Segment ID	SPPS sequence
Ub KtoR **(1)**	MQIFVRTLTGRTITLEVEPSDTIENVRARIQDREGIPPDQQRLIFAGRQLEDGRTLSDYNIQRESTLHLVLRLRGG
FLYRANK **(2)**	FLYRANK
FLYRANK$_{Az}$ **(5)**	FLYRANK$_{Az}$
Ac-FLYRANK **(7)**	ZFLYRANK
K(Ns)LYRANK **(9)**	XLYRANK
Ub (Ns protected) (**11**)	!QIFVXTLTGXTITLEVEPSDTIENVXAXIQDXEGIPPDQQRLIFAGXQLEDGRTLSDYNIQXESTLHLVLRLRGG
Ub (all Ns except K48) (**12**)	!QIFVXTLTGXTITLEVEPSDTIENVXAXIQDXEGIPPDQQRLIFAGKQLEDGRTLSDYNIQXESTLHLVLRLRGG
FUBI	!QLFVRAQELHTFEVTGQETVAQIKAHVASLEGIAPEDQVVLLAGAPLEDEATLGQSGVEALTTLEVAGR!LGG

X = Fmoc-Lys(Ns)-OH, cas: 359780-63-5

! = Fmoc-norleucine-OH, cas: 77284-32-3

Z = acetyl group

Underlined dipeptide sequences were coupled as the respective pseudoproline dipeptides or DMB dipeptides. K$_{Az}$ was incorporated as an azidoleucine. The cysteine of the FUBI sequence is replaced for a serine (coloured orange).

Synthesis of Ac-Ub$_{K\text{-to-}R}$-3,5-dichloro-2-hydroxybenzenesulfonyl ester (1)

The synthesis was performed following general procedures (automated Fmoc SPPS method 2) using 2-chlorotrityl resin preloaded with Gly (0.64 gram, 0.23 mmol/gram). After the synthesis the N-terminus was acetylated using Ac$_2$O (15 eq.) and DIPEA (4 eq.) for 4hr. Test cleavage on an aliquot followed by LC-MS analysis revealed full conversion. The protected protein was detached from the resin according to the general procedures. Subsequently, the protected protein was dissolved in DCM and 3,5-Dichloro-2-hydroxybenzenesulfonyl chloride (1.1 eq., 48 mg, 0.18 mmol) and DIPEA (2

eq., 44 µL, 0.32 mmol) were added, turning the reaction mixture bright yellow. After 15-30 minutes of stirring at r.t a test cleavage on an aliquot followed by LC-MS analysis revealed completion of the reaction. The solvents were removed *in vacuo* and the protecting groups were removed according to the general procedures. The crude protein was purified according to Method 2 using a 30 minute run with a gradient of 5 to 25 % B over 5 minutes, followed by 25 to 35% B over 12 minutes, followed by 25 to 70% B over 8 minutes, followed 70 to 95 % B over 2 minutes. Afterward the pure fractions were pooled and lyophilized to afford peptide **1** as a white solid (22.66 mg, 12.60 % yield).

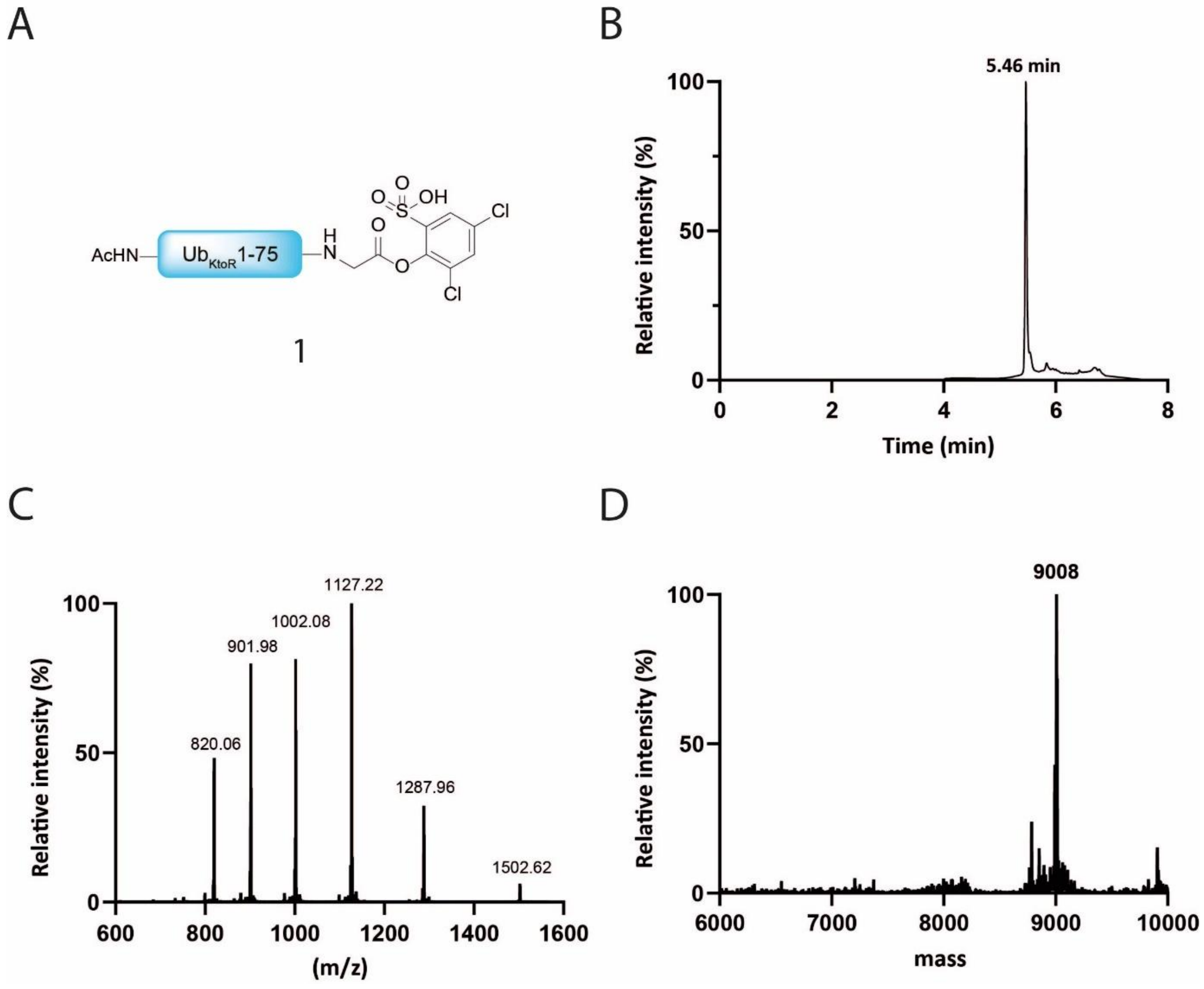

Figure S1. A. Schematic representation of **1**. **B**. Total ion spectrum of purified **1**, Rt 5.46 min (LC-MS method A) **C**. ESI spectrum of purified **1**. Calculated Mass (average isotope composition): 9010.00; $[M + 6H]^{6+}$: 1502.67, $[M + 7H]^{7+}$: 1288,14 $[M + 8H]^{8+}$: 1127.25, $[M + 9H]^{9+}$: 1002.11, $[M + 10H]^{10+}$: 902.00, $[M + 11H]^{11+}$: 820.09. Observed: 9008.00; $[M + 6H]^{6+}$: 1502.62, $[M + 7H]^{7+}$: 1287,96 $[M + 8H]^{8+}$: 1127.22, $[M + 9H]^{9+}$: 1002.08, $[M + 10H]^{10+}$: 901.98, $[M + 11H]^{11+}$: 820.06. **D.** Deconvoluted mass calculated: 9008.00.

Synthesis of H-FLYRANK (2)

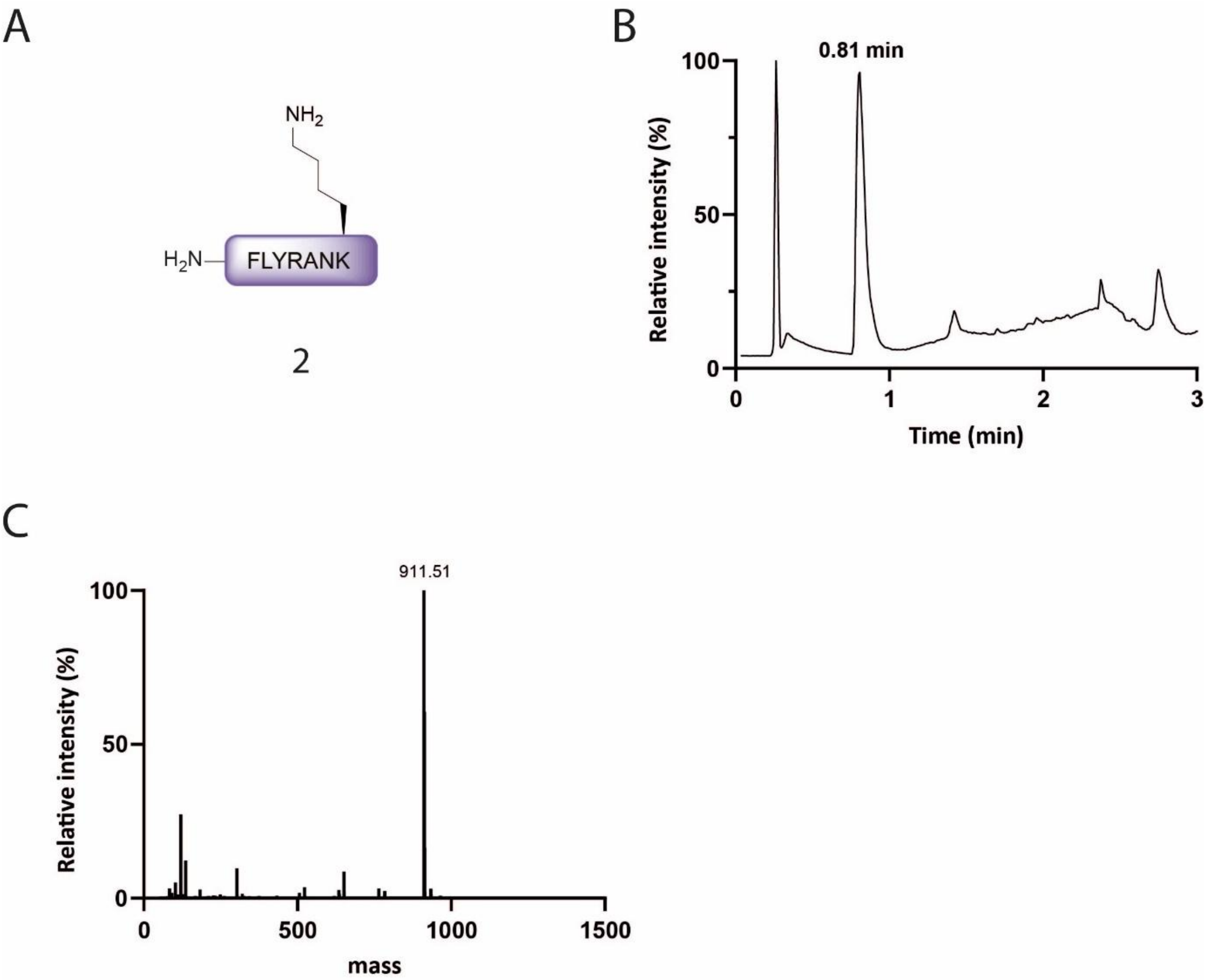

Figure S2. A. Schematic representation of **2**. **B**. Total ion spectrum of purified peptide **2**, Rt 0.81 min, (LC-MS method B). **C**. ESI spectrum of purified peptide **2**. Calculated Mass (average isotope composition): 911.1; $[M + H]^+$: 912.5. Observed: 911.51 ; $[M + H]^+$: 912.55.

The FLYRANK peptide **2** was a kind gift from Jesper Mikkelsen, synthesis as described by Mikkelsen et al.[1]

Synthesis of H-FLYRANK$_{Az}$ (5)

The synthesis was started by manually pre-loading a Wang resin (2.2 mmol/g). Wang-resin was added (45 mg, 0.10 mmol) to an anhydrous DCM/DMF (1:9) solution (1 mL) under a nitrogen atmosphere, resulting in a suspension. In a separate flask, HOBt (34 mg, 2.5 eq.) was added to a solution of Fmoc-L-Lys(N_3)-OH (98.9 mg, 2.5 eq) in anhydrous DMF (0.4 mL) and added to the resin. Pre-dissolved DMAP (1.4 mg, 0.1 eq.) in anhydrous DMF (0.1mL) was subsequently added to the suspension, followed by DIC (5 µL, 2.5 eq.) and the reaction mixture was stirred overnight. Next the reaction mixture was washed using DMF (3x), DCM (3x) and MeOH (3x), and the crude was concentrated under reduced pressure. Afterwards 5 mg of the resin was weighed in a volumetric flask and 20% piperidine/NMP mixture (0.5 mL) was added and was gently shaken for 40 minutes. Then the flask was filled up to 25mL using MeOH and the absorption was measured using a UV-spectrometer at 302 nm, concluding in a loading of 1.48 mmol/g. Afterwards the resin was capped

3x using Ac_2O (95 µL, 10 eq.) and DIPEA (35 µL, 2 eq.) in DMF/DCM (1:1), followed by washing the resin using DMF (4x) and DCM (4x). The peptides were further synthesized following general procedures (automated Fmoc SPPS method 2), followed by cleavage of the protecting groups according to the general procedures. The crude peptide was purified according to Method 2 using a 30 minute run with a gradient of 5 to 25 % B over 5 minutes, followed by 25 to 35% B over 12 minutes, followed by 25 to 70% B over 8 minutes, followed 70 to 95 % B over 2 minutes. Afterward the pure fractions were pooled and lyophilized which afforded peptide **5** as a white solid (0.87 mg, 4.5 % yield).

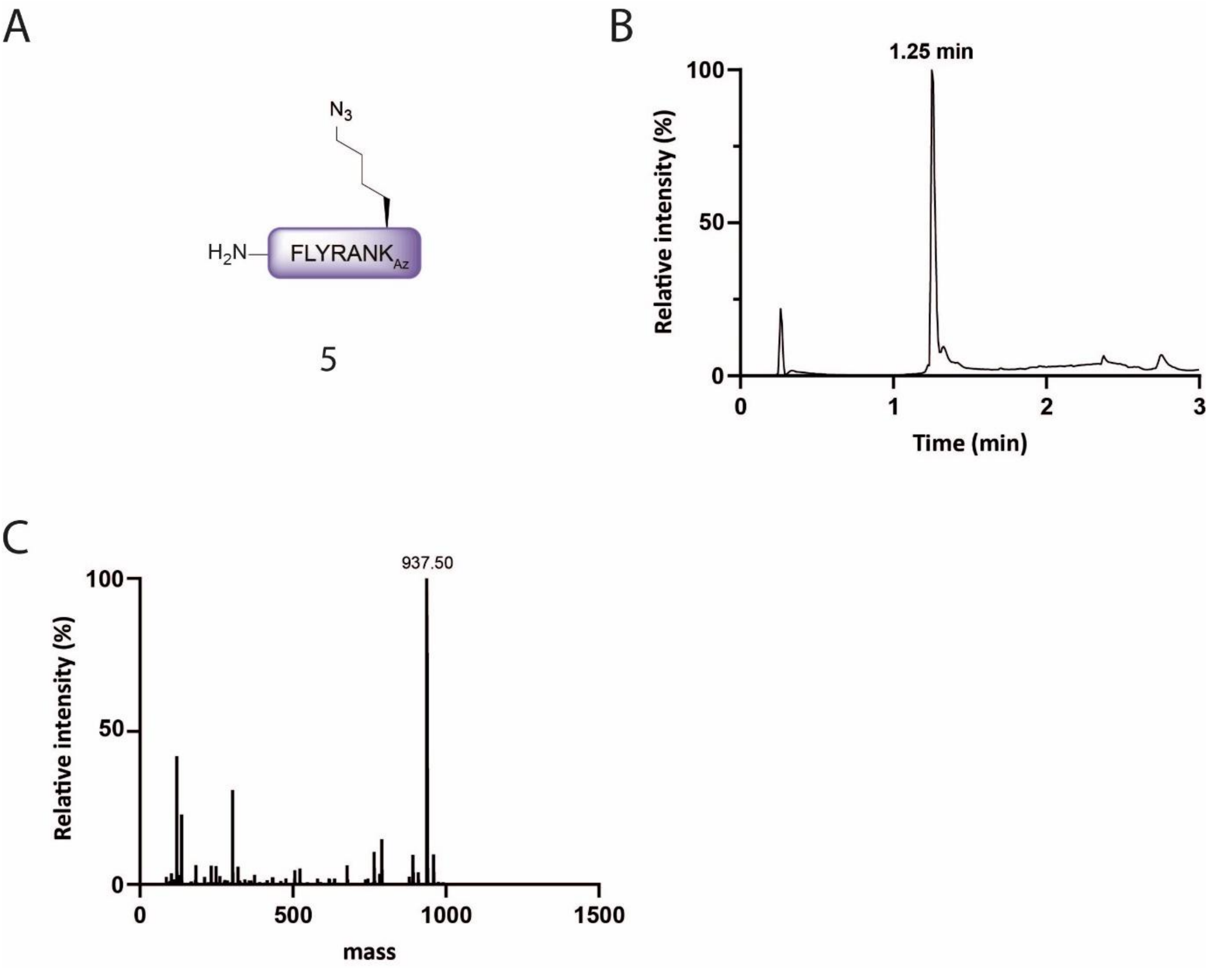

Figure S3. A. Schematic representation of **5**. Total ion spectrum of purified peptide **5**, Rt 1.25 min, (LC-MS method B). **B.** ESI spectrum of purified peptide **5**. C) Calculated Mass (average isotope composition): 937.50; $[M + H]^+$: 938.50. Observed: 937.50 ; $[M + H]^+$: 938.50.

Synthesis of Ac-FLYRANK (7)

The synthesis was performed following general procedures (automated Fmoc SPPS method 2), followed by acylation of the N-terminus using Ac_2O (4 eq.), PYBOP (4 eq.) and DIPEA (8 eq.), for 25 minutes, and was repeated once. Afterwards the peptide was deprotected according to the general procedures. The crude peptide was purified by preparative RP-HPLC using a Phenomenex, Gemini® 110 Å, C18, 5 µm, 30 mm x 250 mm column ((5 to 20 % B over 30 min, 30 mL/min) followed by lyophilization to afford peptide **7** as a white solid (4.4 mg, 24.2 % yield).

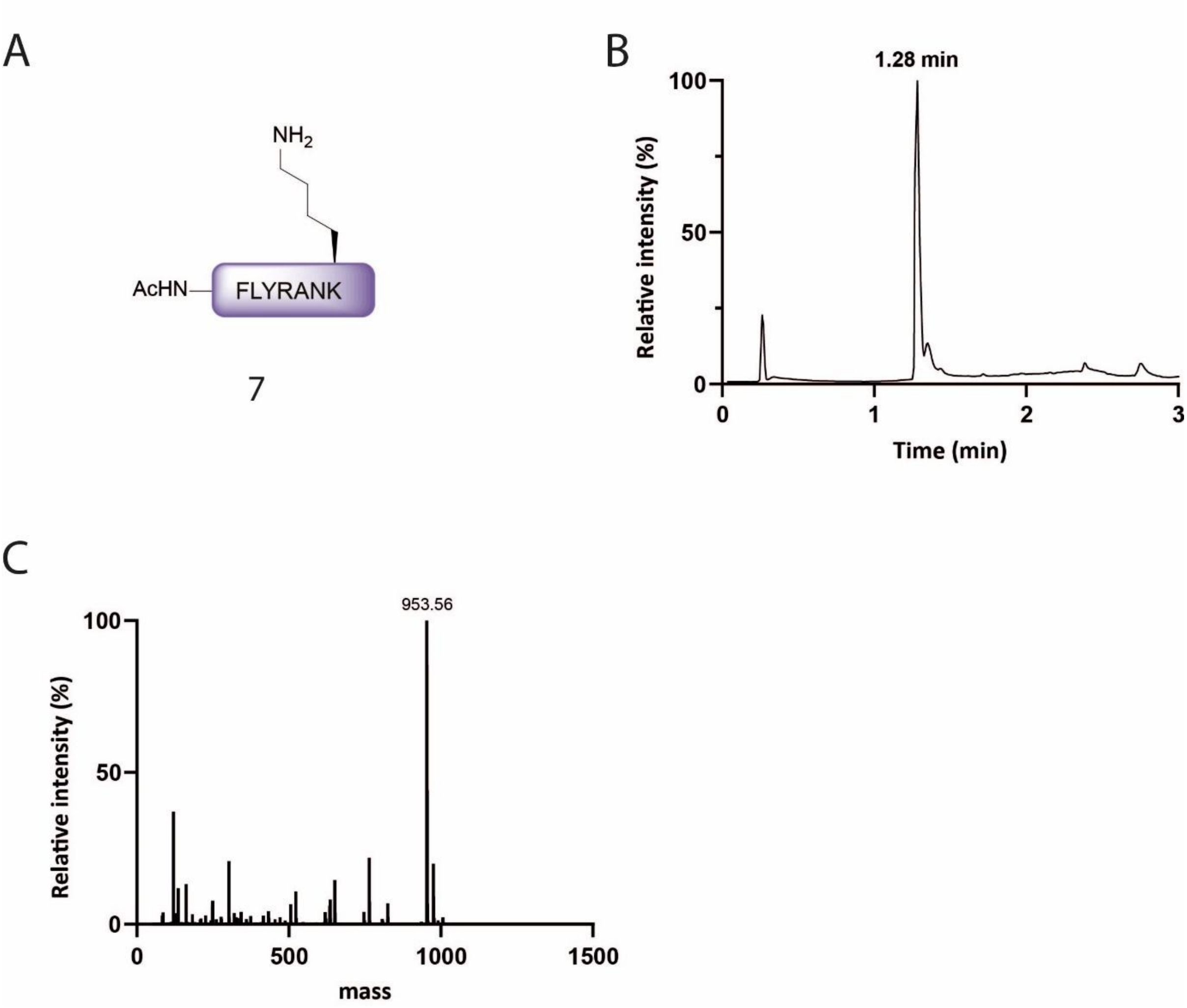

Figure S4. A. Schematic representation of **7**. Total ion spectrum of purified peptide **7**, Rt 1.28 min, (LC-MS method B). **B.** ESI spectrum of purified peptide **7**. Calculated Mass (average isotope composition): 953.5; $[M + H]^+$: 954.50. Observed: 953.56 ; $[M + H]^+$: 954.56.

Synthesis of H-K(Ns)LYRANK (9)

The synthesis was performed following general procedures (automated Fmoc SPPS method 2) using preloaded Wang Lysine resin (105 mg, 0.19 mmol/gram). The peptide was cleaved from the resin according to the general procedures and purified by preparative RP-HPLC using a Phenomenex, Gemini® 110 Å, C18, 5 µm, 30 mm x 250 mm column (5 to 20 % B over 30 min, 30mL/min) followed by lyophilization to afford peptide **9** as a white solid (7.1 mg, 33.1% yield).

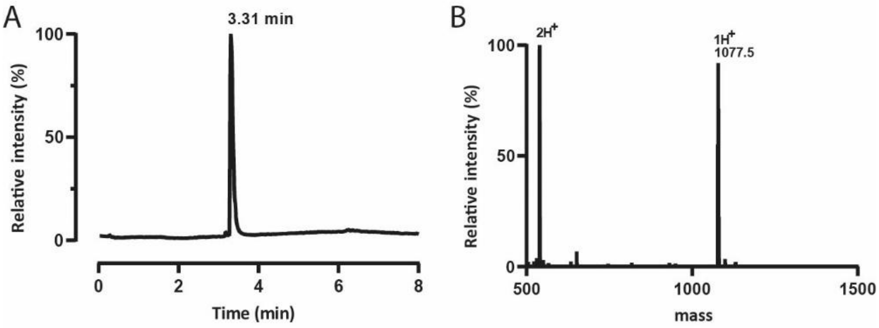

Figure S5. A. Total ion spectrum (LC-MS method C) of purified **9**, Rt 3.31 min. **B.** ESI spectrum of purified **9**. Calculated Mass (average isotope composition): 1076.52; $[M + H]^+$: 1077.52, $[M + 2H]^{2+}$: 539.26. Observed: 1076.58 ; $[M + H]^+$: 1077.58, $[M + 2H]^{2+}$: 539.30.

Synthesis of Ub (all lysine Ns-protected) activated ester (11)

The synthesis was performed following general procedures (automated Fmoc SPPS method 2) using 2-chlorotrityl resin preloaded with Gly (0.11 gram, 0.17 mmol/gram). After the synthesis the N-terminus was boc protected using Boc_2O (10 eq.) and DIPEA (2 eq.). The protected peptide was detached from the resin according to the general procedures. Subsequently, the protected protein was dissolved in DCM and 3,5-dichloro-2-hydroxybenzenesulfonyl chloride (1.1 eq., 5.6 mg, 0.02 mmol) and DIPEA (2 eq. 6.5 µL, 0.04 mmol) were added, upon which the reaction turned bright yellow. After 15-30 minutes the reaction turned pale yellow and a test cleavage on an aliquot followed by LC-MS analysis revealed completion of the reaction. The solvents were removed *in vacuo* and the protecting groups were removed according to the general procedures. The crude peptide was purified by preparative RP-HPLC using a Phenomenex, Gemini® 110 Å, C18, 5 µm, 30 mm x 250 mm column (20 to 45 % B over 30 min, 30mL/min) followed by lyophilization afforded peptide **11** as a white solid (4.7 mg, 4.7 % yield).

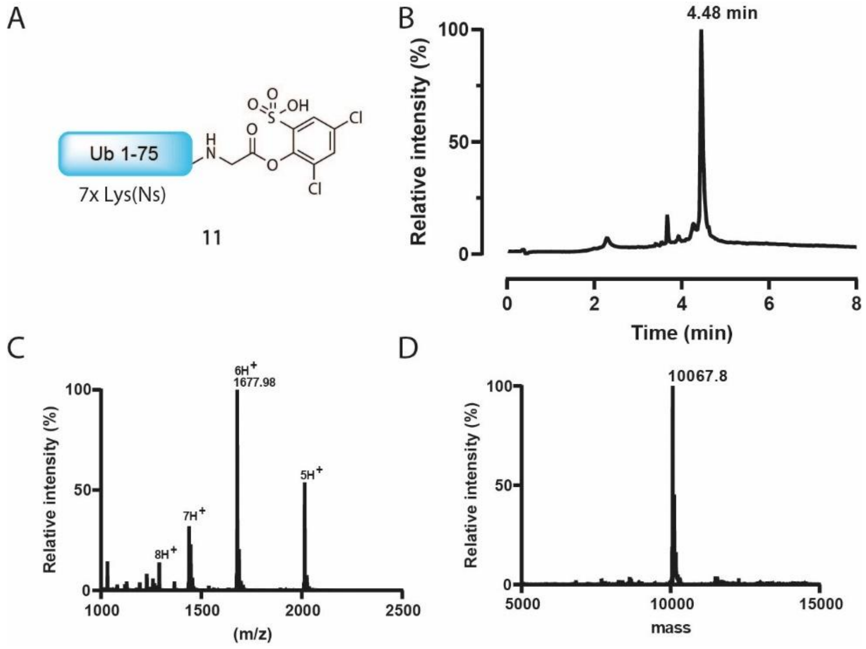

Figure S6. A. Schematic representation of **11**. **B.** Total ion spectrum (LC-MS method D) of purified **11**, Rt 4.48 min. **C.** ESI spectrum of purified **11**. Calculated Mass (average isotope composition): 10060.42; $[M + 5H]^{5+:}$ 2013.08 $[M + 6H]^{6+}$: 1677.74, $[M + 7H]^{7+}$: 1438.20, $[M + 8H]^{8+}$: 1258.55. Observed: 10061.8; $[M + 5H]^{5+}$: 2013.36, $[M + 6H]^{6+}$: 1677.98, $[M + 7H]^{7+}$: 1438.20, $[M + 8H]^{8+}$: 1258.75. **D.** Deconvoluted mass calculated: 10067.8, Observed: 10067.8.

Synthesis of Ub (all lysine Ns-protected except K48) (12)

The synthesis was performed following general procedures (automated Fmoc SPPS method 2) using 2-chlorotrityl resin preloaded with Gly (0.05 gram, 0.17 mmol/gram). The peptide was cleaved from the resin according to the general procedures and purified by preparative RP-HPLC using a Phenomenex, Gemini® 110 Å, C18, 5 µm, 30 mm x 250 mm column (20 to 45 % B over 30 min, 30mL/min) followed by lyophilization afforded peptide **12** as a white solid (6.1 mg, 6.3% yield).

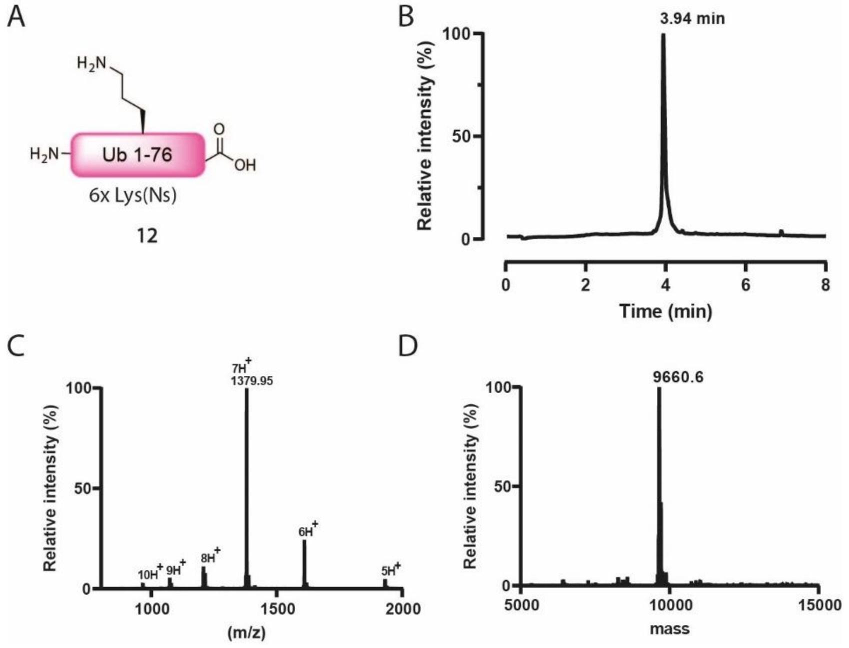

Figure S7. A. Schematic representation of **12**. **B.** Total ion spectrum (LC-MS method D) of purified **12**, Rt 3.94 min. **C.** ESI spectrum of purified **12**. Calculated Mass (average isotope composition): 9651.53; $[M + 5H]^{5+}$: 1931.31 $[M + 6H]^{6+}$: 1609.59, $[M + 7H]^{7+}$: 1379.79, $[M + 8H]^{8+}$: 1207.44, $[M + 9H]^{9+}$: 1073.39. Observed: 9652.5; $[M + 5H]^{5+}$: 1931.50, $[M + 6H]^{6+}$: 1609.77, $[M + 7H]^{7+}$: 1379.95, $[M + 8H]^{8+}$: 1207.58, $[M + 9H]^{9+}$: 1073.41. **D.** Deconvoluted mass calculated: 9657.6, Observed: 9660.6

Ubiquitination with activated phenol esters

Acylation of peptide 2 with Ub 1 at pH 10.5.

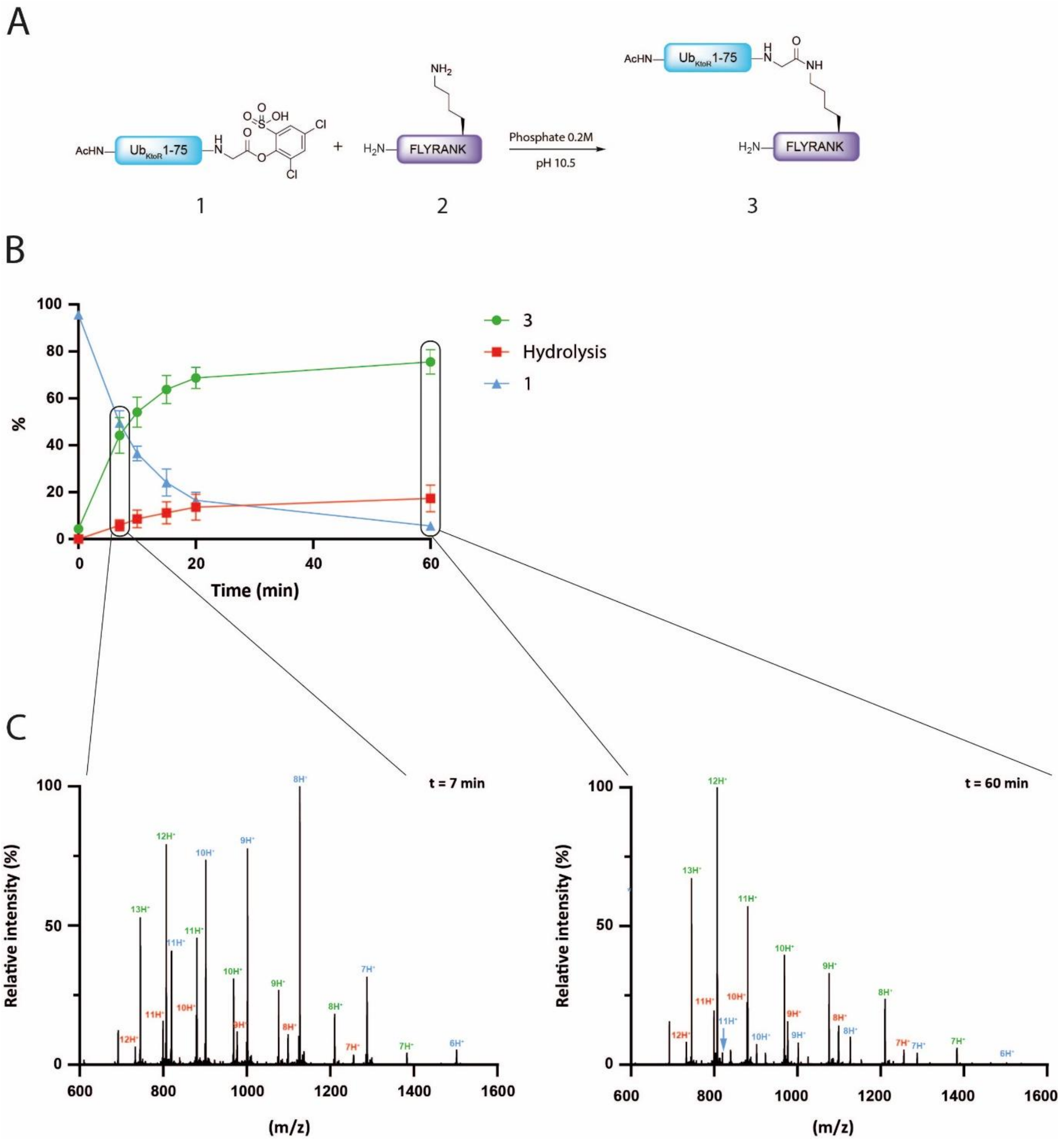

Figure S8. A. Reaction scheme of the acylation of peptide **2** by activated Ub **1** at pH 10.5**. B.** Formation of Ub$_{KtoR}$-FLYRANK **3** (light green line), Ub-ester **1** hydrolysis (red line) and the remaining starting material **1** (blue line) as relative percentage. **C.** Total Ion Spectra (LC-MS Method A) of compound **3** at t = 7 minutes (min) at t = 60 minutes (min). Blue = Ub **1**, red = hydrolysis of Ub **1**, green = compound **3**. Calculated Mass (average isotope composition) of **3**: 9678.03 $[M + 7H]^{7+}$: 1383.58,

$[M + 8H]^{8+}$: 1210.75, $[M + 9H]^{9+}$: 1076.34, $[M + 10H]^{10+}$: 968.80, $[M + 11H]^{11+}$: 880.82, $[M + 12H]^{12+}$: 807.50, $[M + 13H]^{13+}$: 745.46. Observed: 9678.95; $[M + 7H]^{7+}$: 1383.47, $[M + 8H]^{8+}$: 1210.66, $[M + 9H]^{9+}$: 1076.26, $[M + 10H]^{10+}$: 968,73 $[M + 11H]^{11+}$: 880.75, $[M + 12H]^{12+}$: 807.45, $[M + 13H]^{13+}$: 745.41. **C.** Deconvoluted mass calculated: 9678.03, Observed: 9678.00.

Peptide **2** (0.11 mg, 5 eq.) was dissolved in freshly prepared phosphate buffer containing 0.2 M Na_2HPO_4, 0.2M NaH_2PO_4 at pH 10.5 (20 µL). Next, activated ester **1** (0.21 mg, 1 eq.) was dissolved in DMSO (10 µL) and was added at a final concentration of 0.75 mM. The pH of the resulting solution was re-adjusted to pH 10.5 and shaken at 350 rpm for 60 minutes at 25 °C. Samples (0.5 µL diluted in 200 µL MQ, adjusted to acidic pH to quench the reaction) were taken at t=7 min, t=10 min, t=15 min, t=20 min and t=60 min and measured using High Resolution Mass Spectrometry (Method A).

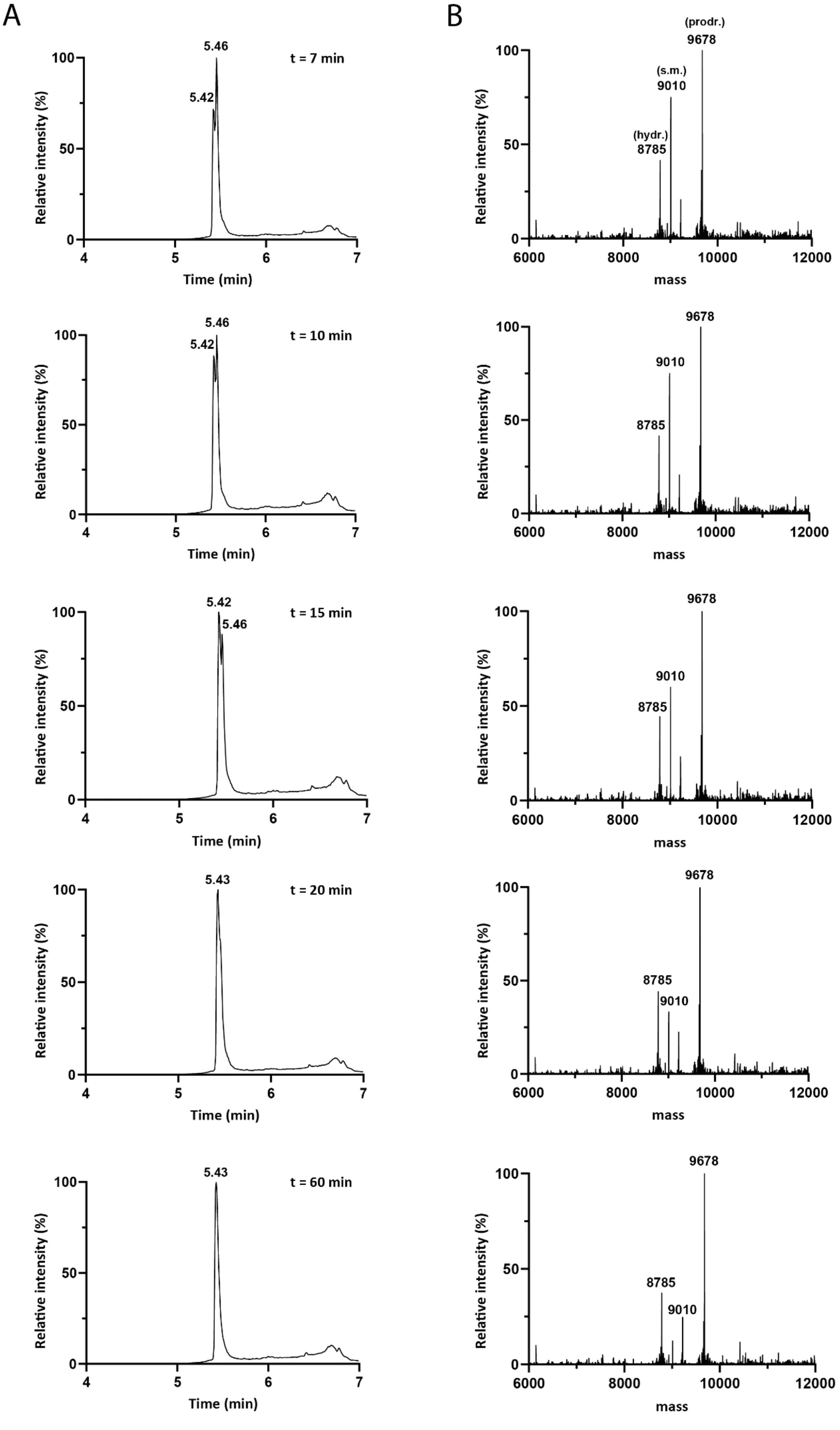

Figure S9. Ubiquitination of peptide **2** with activated Ub **1** followed over a period of one hour. **A**. Total ion spectra of the subsequent timepoints. **B**. Deconvoluted masses of peptide **3**: calculated: 9678.0, Observed: 9678.0. The mass 8785 is hydrolyzed Ub **1**.

Acylation of peptide 2 with Ub 1 at pH 7.4.

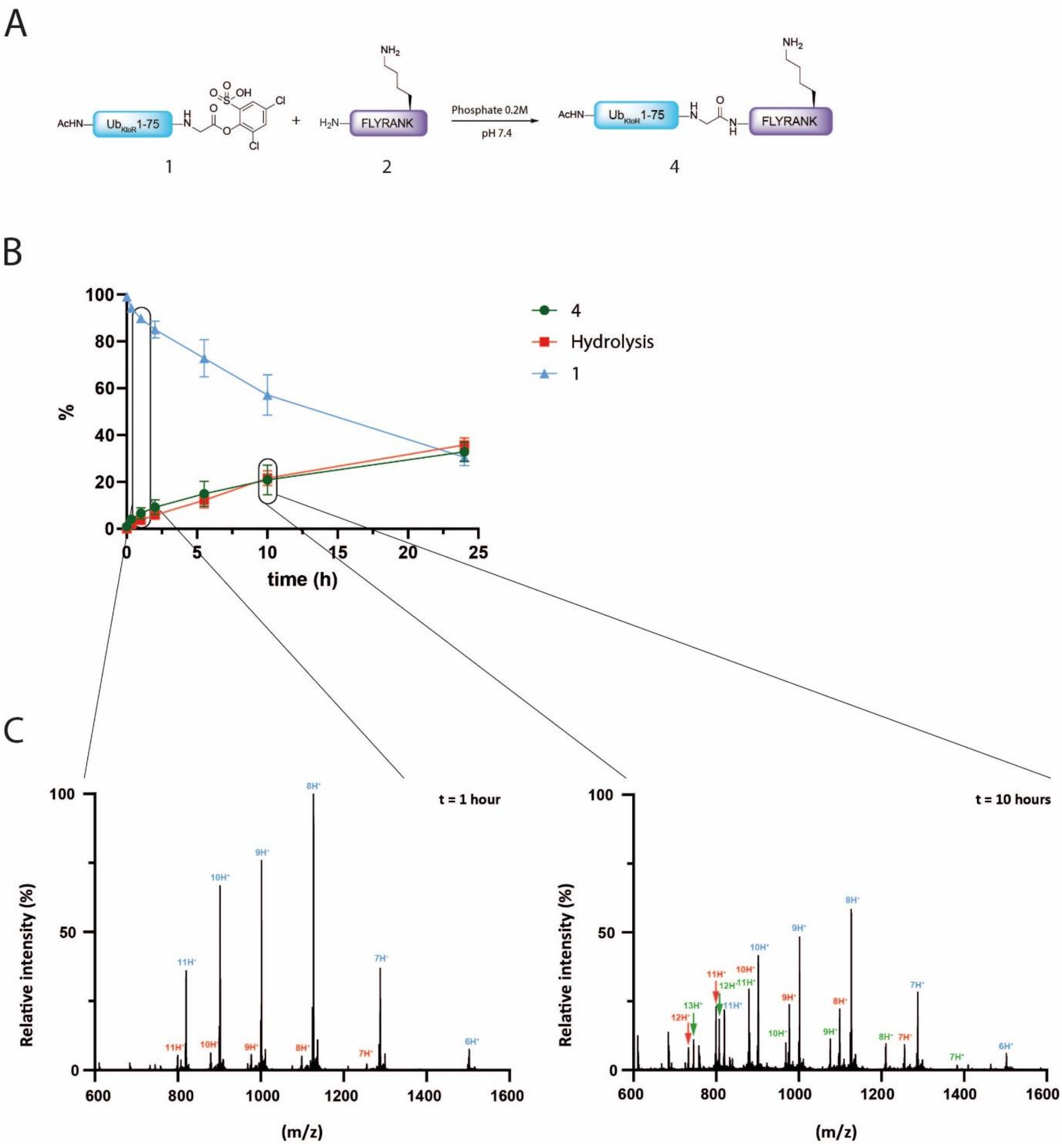

Figure S10. A. Reaction scheme of the acylation of peptide **2** by activated Ub **1** at pH 7.4**. B.** Formation of Ub_{KtoR}-FLYRANK **4** (dark green line), Ub-ester **1** hydrolysis (red line) and remaining starting material **1** (blue line) as relative percentage. **C.** Total Ion Spectra (LC-MS Method A) of compound **3** at t = 1 hour at t = 10 hours. Blue = Ub **1**, red = hydrolysis of Ub **1**, green = compound **3**. Calculated Mass (average isotope composition) of **3**: 9678.03 $[M + 7H]^{7+}$: 1383.58, $[M + 8H]^{8+}$: 1210.75, $[M + 9H]^{9+}$: 1076.34, $[M + 10H]^{10+}$: 968.80, $[M + 11H]^{11+}$: 880.82, $[M + 12H]^{12+}$: 807.50, $[M + 13H]^{13+}$: 745.46. Observed: 9678.95; $[M + 7H]^{7+}$: 1383.47, $[M + 8H]^{8+}$: 1210.66, $[M + 9H]^{9+}$: 1076.26, $[M + 10H]^{10+}$: 968,73 $[M + 11H]^{11+}$: 880.75, $[M + 12H]^{12+}$: 807.45, $[M + 13H]^{13+}$: 745.41. **C.** Deconvoluted mass calculated: 9678.03, Observed: 9678.00.

Peptide **2** (0.11 mg, 5 eq.) was dissolved in freshly prepared phosphate buffer containing 0.2 M Na_2HPO_4, 0.2M NaH_2PO_4 at pH 7.4 (20 µL). Next, activated ester **1** (0.21 mg, 1 eq.) was dissolved in DMSO (10 µL) and added at a final concentration of 0.75 mM. The resulting solution was adjusted to pH 7.4 again and was shaken at 350 rpm for 24 hours at 25 °C. Samples (0.5 µL in 200 µL MQ, adjusted to acidic pH to quench the reaction) were taken at t=20 minutes, t=1hr, t=2hr, t=5.5hr and t=10hr and t=24hr and were measured using High Resolution Mass Spectrometry.

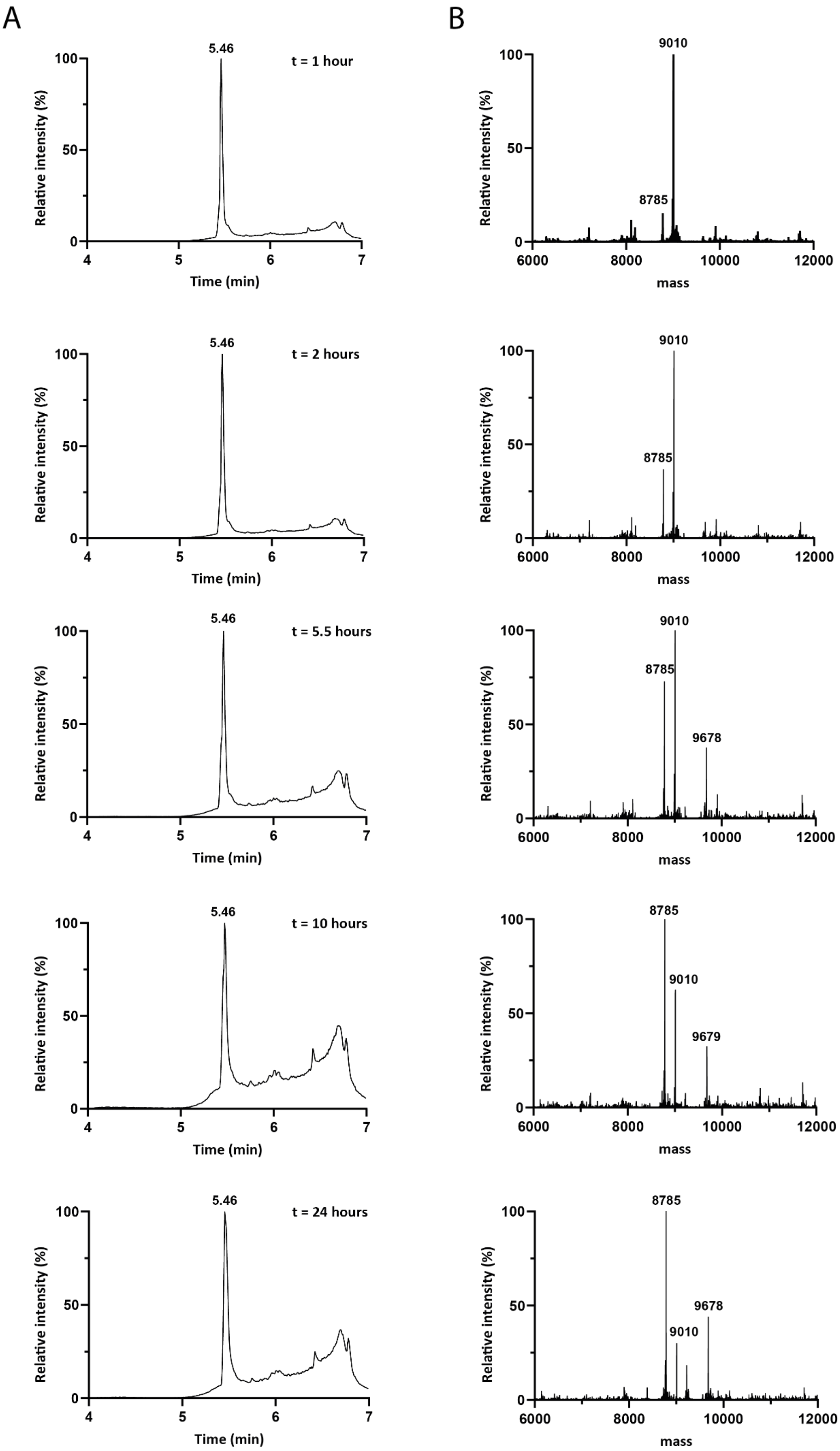

Figure S11. Ubiquitination of peptide **4** with activated Ub ester **1** followed over a period of 24 hours. **A**. Total ion spectra. **B**. Deconvoluted mass of peptide **4**: calculated: 9678.0, Observed: 9678.0. The mass 8785 is hydrolyzed Ub ester **1**

Acylation of peptide 5 with Ub 1 at pH 10.5.

The same method as for the ubiquitination of peptide **2** was executed.

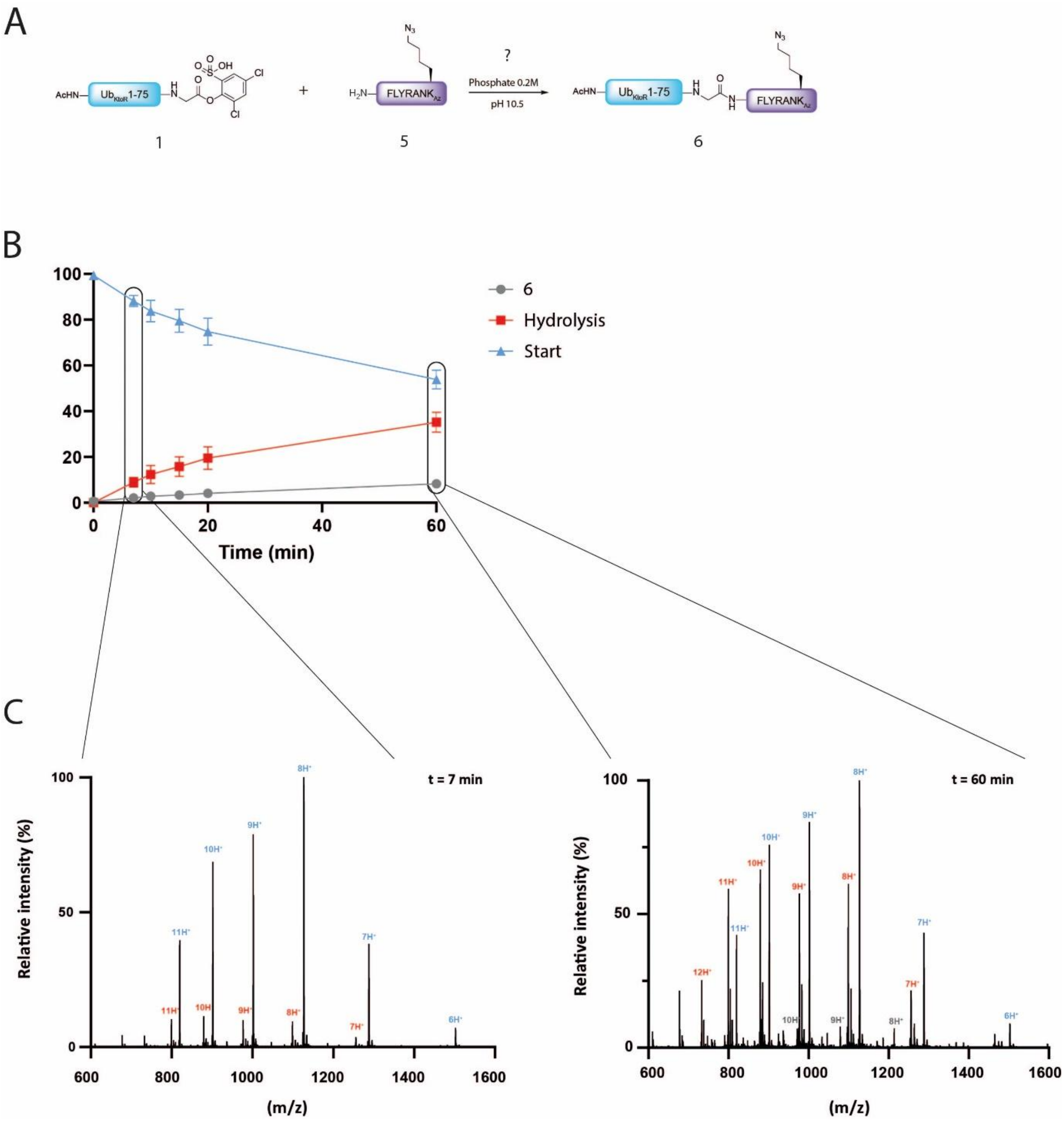

Figure S12. A. Reaction scheme of the acylation of peptide **5** by activated Ub **1** at pH 10.5**. B.** Formation of Ub$_{KtoR}$-FLYRANK$_{Az}$ **6** (gray line), Ub-ester **1** hydrolysis (red line) and remaining starting material **1** (blue line) as relative percentage. **C.** Total Ion Spectra (LC-MS Method A) of compound **6** at t = 7 minutes (min) and at t = 60 minutes (min). Blue = Ub **1**, red = hydrolysis of Ub **1**, gray = compound **6**. Calculated Mass (average isotope composition) of **6**: 9705.05 $[M + 7H]^{7+}$: 1387.43, $[M + 8H]^{8+}$: 1214.13, $[M + 9H]^{9+}$: 1079.33, $[M + 10H]^{10+}$: 971.50, $[M + 11H]^{11+}$: 883.27, $[M + 12H]^{12+}$: 809.75, $[M + 13H]^{13+}$: 747.54. Observed: 9704.00; $[M + 7H]^{7+}$: 1387.18, $[M + 8H]^{8+}$: 1213.91, $[M + 9H]^{9+}$: 1079.15, $[M + 10H]^{10+}$: 971,32 $[M + 11H]^{11+}$: 883.12, $[M + 12H]^{12+}$: 809.69, $[M + 13H]^{13+}$: 747.41. **C.** Deconvoluted mass calculated: 9705.05, Observed: 9704.00. For the sake of clarity, only the charge states $[M + 10H]^{10}$, $[M + 9H]^{9+}$, $[M + 8H]^{8+}$ are shown in the graph.

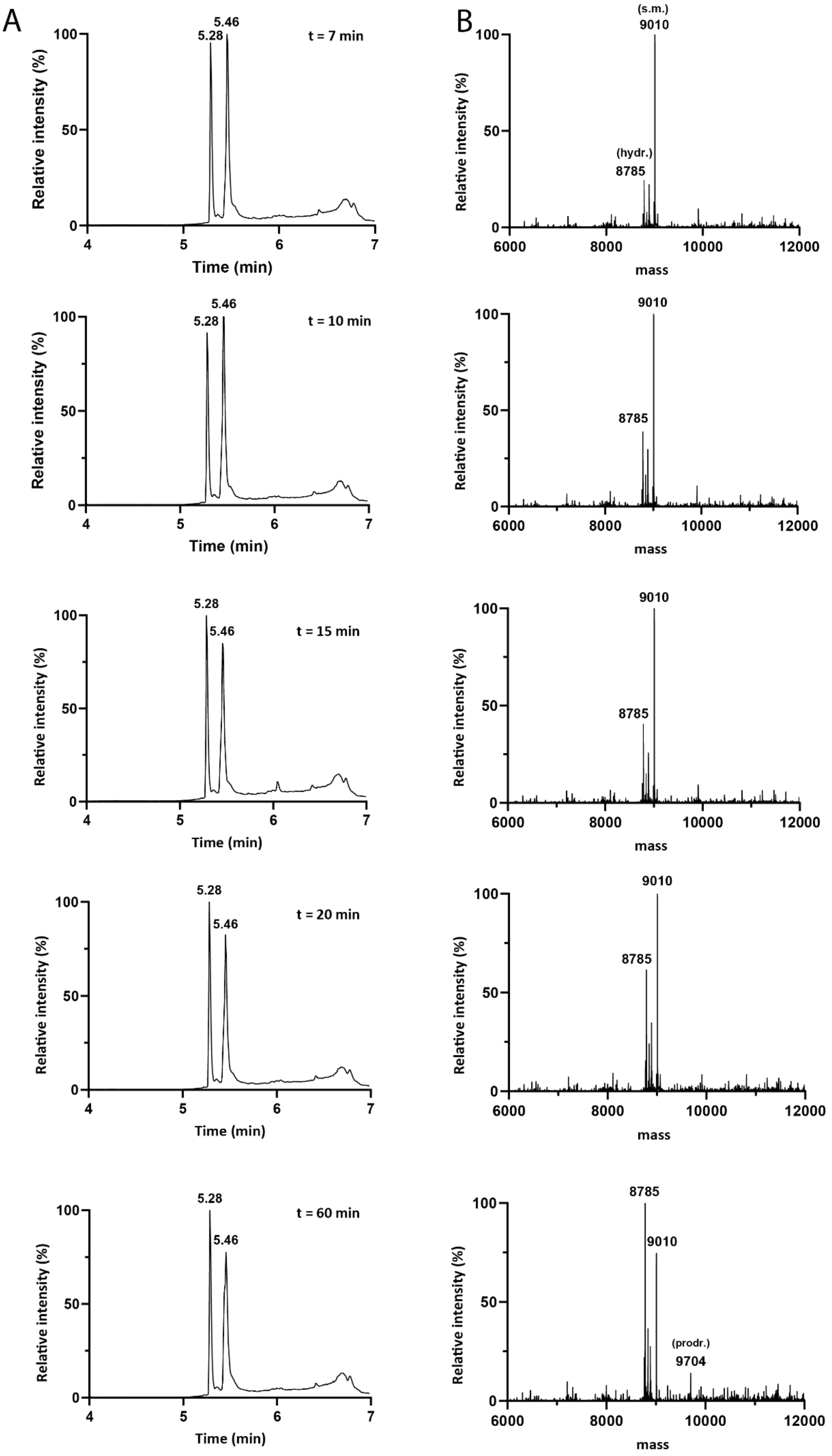

Figure S13. Ubiquitination of peptide **5** with activated Ub ester **1** followed over a period of 60 minutes (min). **A**. Total ion spectra. **B**. Deconvoluted mass of peptide **6**: calculated: 9704.0, Observed: 9704.0. The mass 8785 is hydrolyzed Ub ester **1**.

Acylation of peptide 7 with Ub 1 at pH 7.4.

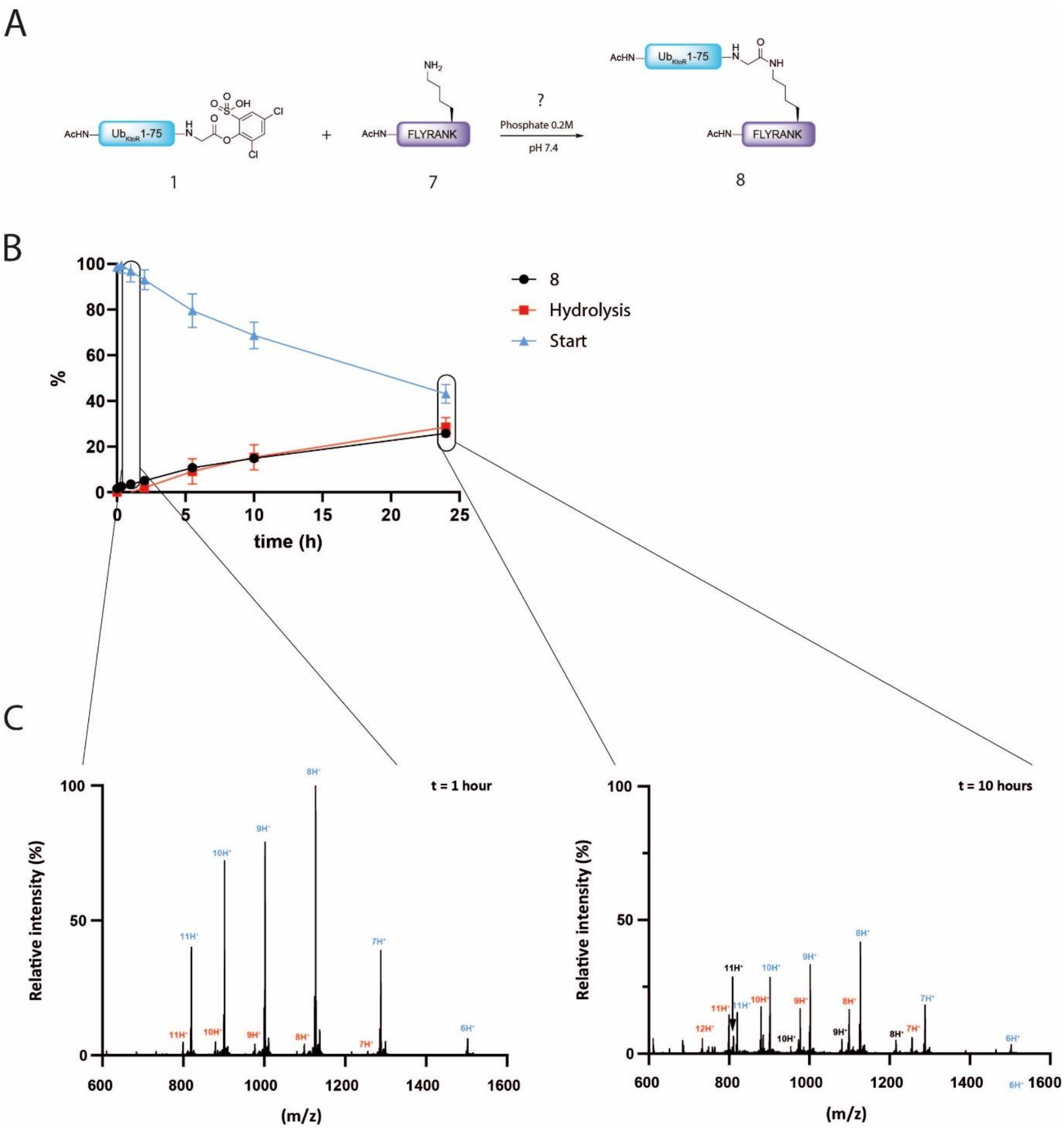

Figure S14. A. Reaction scheme of the acylation of peptide **7** by activated Ub **1** at pH 10.5**. B.** Formation of Ub_{KtoR}-Ac-FLYRANK **8** (gray line), Ub-ester **1** hydrolysis (red line) and remaining starting material **1** (blue line) as relative percentage. **C.** Total Ion Spectra (LC-MS Method A) of compound **6** at t = 1 hour and at t = 7 hours. Blue = Ub **1**, red = hydrolysis of Ub **1**, black = compound **8**. Calculated Mass (average isotope composition) of **8**: 9720.07 $[M + 7H]^{7+}$: 1389.57, $[M + 8H]^{8+}$: 1216.00, $[M + 9H]^{9+}$: 1081.00, $[M + 10H]^{10+}$: 973.00, $[M + 11H]^{11+}$: 884.64, $[M + 12H]^{12+}$: 811.00, $[M + 13H]^{13+}$: 748.69. Observed: 9720.00; $[M + 7H]^{7+}$: 1389.62, $[M + 8H]^{8+}$: 1215.91, $[M + 9H]^{9+}$: 1081.03, $[M + 10H]^{10+}$: 972,93 $[M + 11H]^{11+}$: 884.67, $[M + 12H]^{12+}$: 811.03, $[M + 13H]^{13+}$: 748.79. **C.** Deconvoluted mass calculated: 9720.07, Observed: 9720.00. For the sake of clarity, only the charge states $[M + 11H]^{11+}$, $[M + 10H]^{10+}$, $[M + 9H]^{9+}$, $[M + 8H]^{8+}$ are shown in the graph.

The same method as for the acylation of peptide **5** was executed.

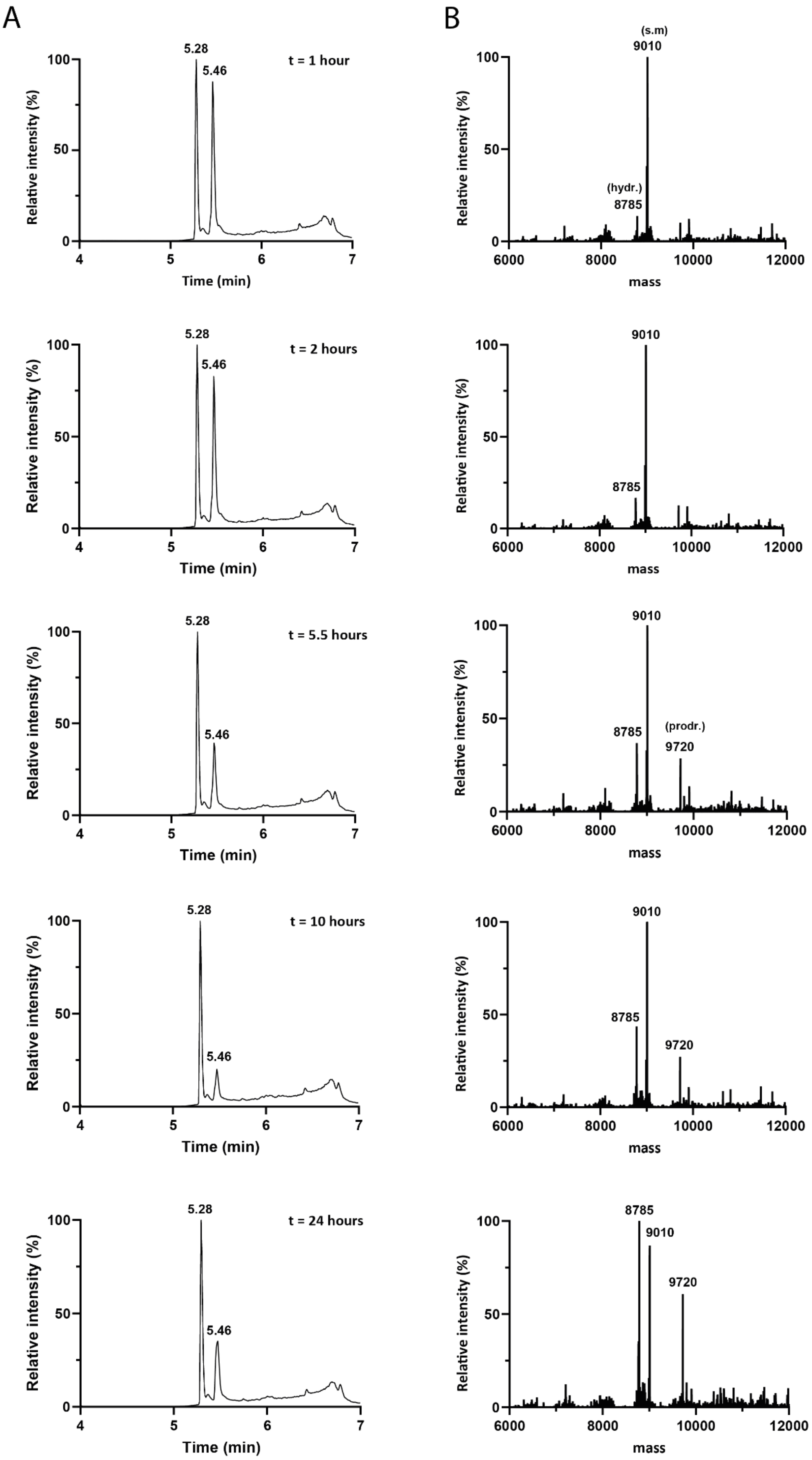

Figure S15. Ubiquitination of peptide **7** with activated Ub ester **1** followed over a period of 24 hours. **A**. Total ion spectra. **B**. Deconvoluted mass of peptide **7**: calulated: 9720.0, Observed: 9720.0. The mass 9010 is Ub ester **1**, mass 8785 is hydrolyzed Ub ester **1**.

Ubiquitination of peptide 4 with peptide 1A and nosyl deprotection

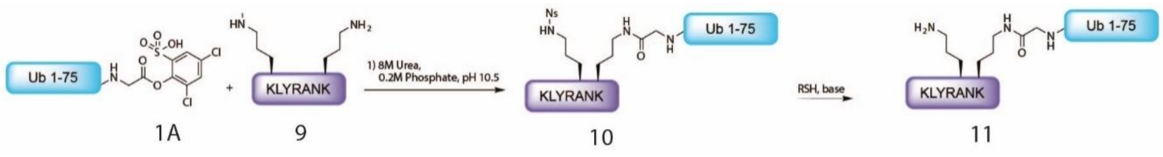

Scheme S1. Reaction scheme of the acylation of peptide **9** by activated Ub **1A.**

Peptide **9** (1.2 equiv.) was dissolved in freshly prepared 8 M Urea containing 0.2 M Na_2HPO_4 at pH 10.5. Next, the activated ester **1A** (1 equiv.) was added as solid to this mixture at a final concentration of 0.5 mM – 1 mM. The resulting solution was shaken (350 rpm) at room temperature for 15 minutes. Samples were taken at t = 1 and t = 10 minutes and analyzed by LC-MS (Fig. S16). After the acylation had reached completion the pH of the solution was adjusted to pH 7.4, followed by the addition of 4-mercaptophenylacetic acid (MPAA) (100 eq.) and K_2CO_3 (2 eq.) both from a 1 M stock solution in MilliQ. The reaction mixture was shaken (350 rpm) at room temperature for 16 hours. LC-MS analysis showed no full conversion, therefore the reaction mixture was heated to 40 °C for an additional 4 hours until the reaction was completed as judged using LC-MS (Fig. S17).

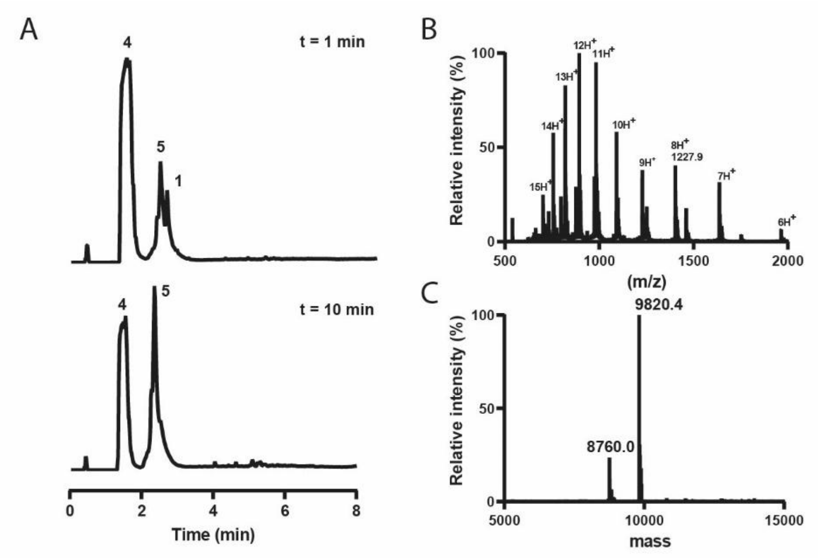

Figure S16. A. Ubiquitination of peptide **9** at t = 1 min and t = 10 min. Nosyl deprotection after the acylation of K(Ns)LYRANK with **1**. **B.** Total ion spectrum of **10**: Calculated Mass (average isotope composition): 9814.15; $[M + 5H]^{5+}$: 1963.83, $[M + 6H]^{6+}$: 1636.69, $[M + 7H]^{7+}$: 1403.02, $[M + 8H]^{8+}$: 1227.77, $[M + 9H]^{9+}$: 1091.46, $[M + 10H]^{10+}$: 982.41, $[M + 11H]^{11+}$: 893.19, $[M + 12H]^{12+}$: 818.85, $[M + 13H]^{13+}$: 755.93. Observed: 9815.1; $[M + 5H]^{5+}$: 1964.02, $[M + 6H]^{6+}$: 1636.83, $[M + 7H]^{7+}$: 1403.15, $[M + 8H]^{8+}$: 1227.89, $[M + 9H]^{9+}$: 1091.56, $[M + 10H]^{10+}$: 982.53, $[M + 11H]^{11+}$: 893.29. **C.** Deconvoluted mass calculated: 9820.0, Observed: 9820.0.

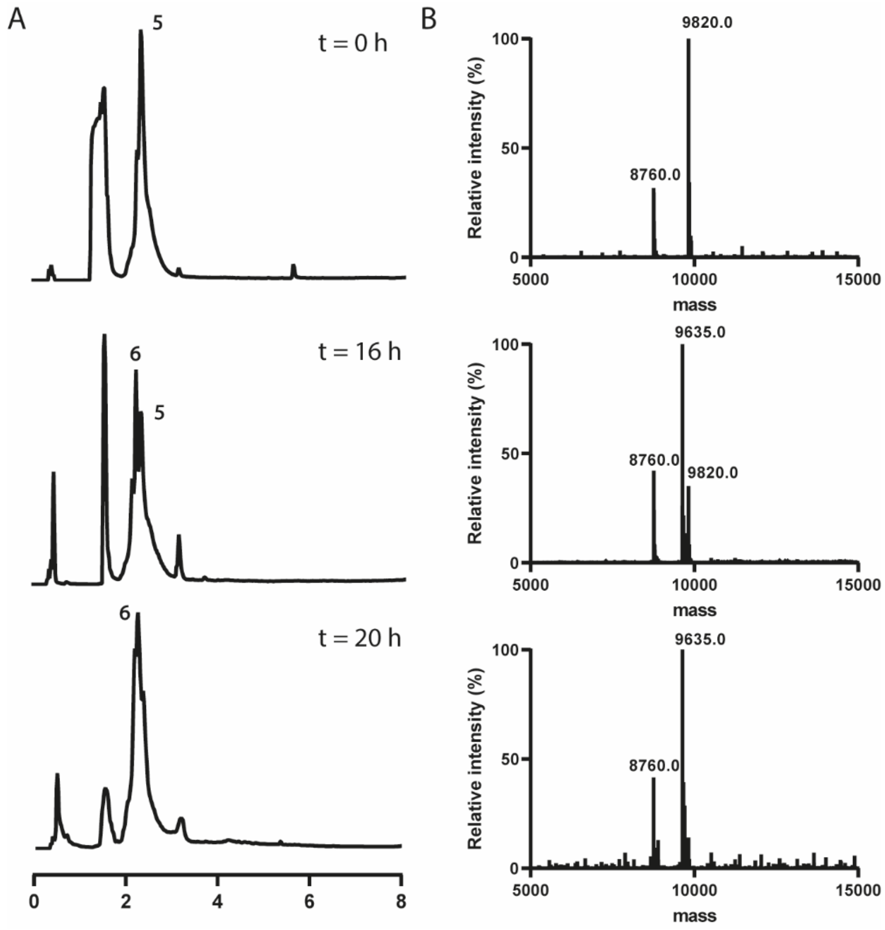

Figure S17. A.) Nosyl deprotection after the acylation of H-K(Ns)LYRANK with **1A** over a time period of 20 hours. **B.)** Deconvoluted mass of peptide **10**: calculated: 9820.0, Observed: 9820.0. Mass of peptide **11**: Calculated Mass (average isotope composition): 9629.17; $[M + 6H]^{6+}$: 1605.86, $[M + 7H]^{7+}$: 1376.60, $[M + 8H]^{8+}$: 1204.65, $[M + 9H]^{9+}$: 1070.91, $[M + 10H]^{10+}$: 963.92, $[M + 11H]^{11+}$: 876.38, $[M + 12H]^{12+}$: 803.43, $[M + 13H]^{13+}$: 741.71. Observed: 9629.94; $[M + 6H]^{6+}$: 1605.99, $[M + 7H]^{7+}$: 1376.72 $[M + 8H]^{8+}$: 1204.65, $[M + 9H]^{9+}$: 1071.00, $[M + 10H]^{10+}$: 964.00, $[M + 11H]^{11+}$: 876.46. **D.** Deconvoluted mass calculated: 9634.9, Observed: 9635.0.

Polymerization of reactions of activated Ub 12.

The peptide was dissolved in buffer containing 0.2 M Na_2HPO_4, at pH 10.5 or 7.4 at a concentration of 0.5 - 1 mM. The reaction mixture was shaken at 350 rpm and 2 µg protein was taken out of the reaction mixture at the indicated time points. The reaction is resolved on 4-12% SDS-page gradient gels (Invitrogen) run in MES buffer (Invitrogen) and stained by Instant Blue (Abcam) protein stain.

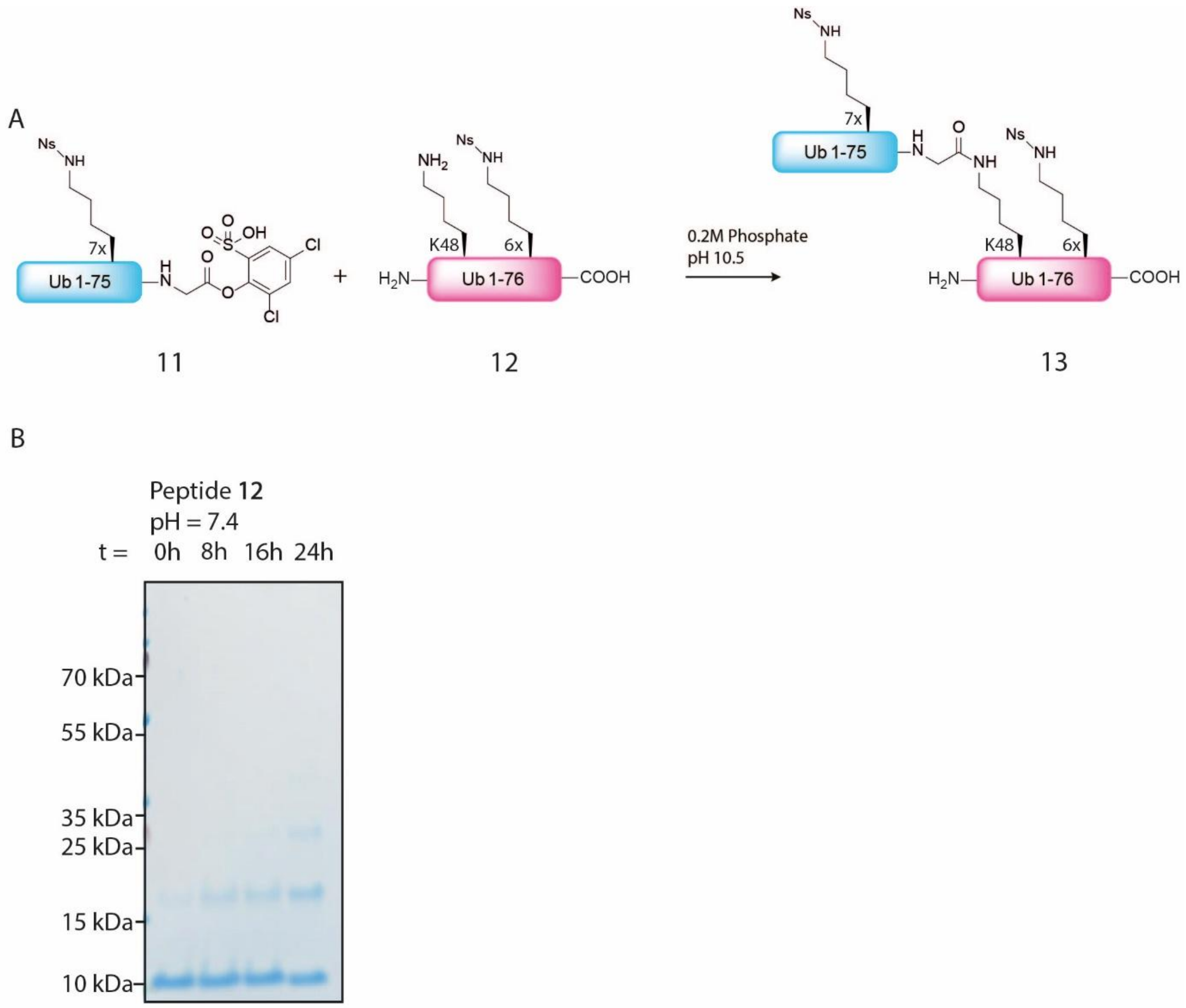

Figure S18. A. Schematic representation of synthesized Ub derivatives **11** and **12** **B.** Self-conjugation of activated Ub **12** at pH 7.4.

Acylation of FUBI with Ub 1 at pH 10.5.

FUBI (**9**) (0.23 mg, 1 eq) was dissolved in freshly prepared urea phosphate buffer containing 8M Urea, 0.2 M Na_2HPO_4 and 0.2M NaH_2PO_4 at pH 10.5 (70 µL) and was stirred at r.t at 350 rpm for 15 minutes. Next, activated ester **1** (0.52 mg, 2 eq.) was dissolved in DMSO (8 µL) and was added slowly to the FUBI solution at a final concentration of 0.31mM. The resulting solution was adjusted to pH 10.5 again and was shaken at 350 rpm at 25°C for 24 hours. Samples (2 µL in 18 µL MQ, ~pH 2) were taken at t=0 min, t=5 minutes, t=10 minutes, t=15 minutes, t=20 minutes and t=60 minutes and t=overnight. All samples were subjected to SDS-page gel analysis.

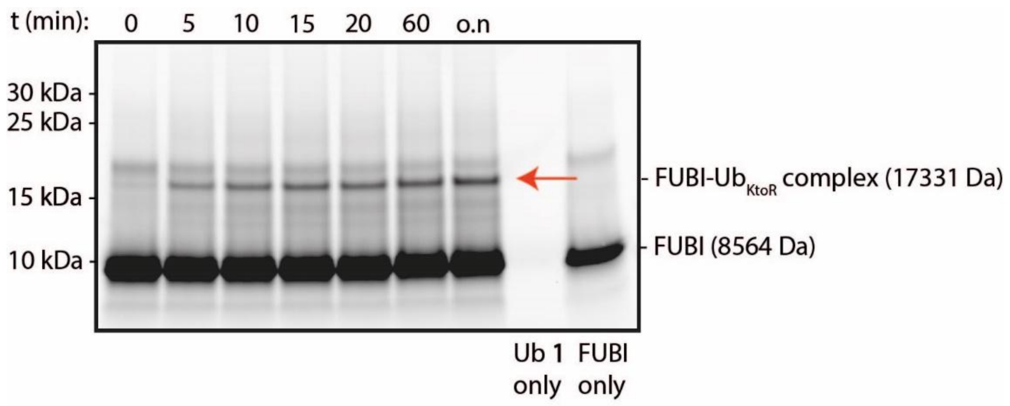

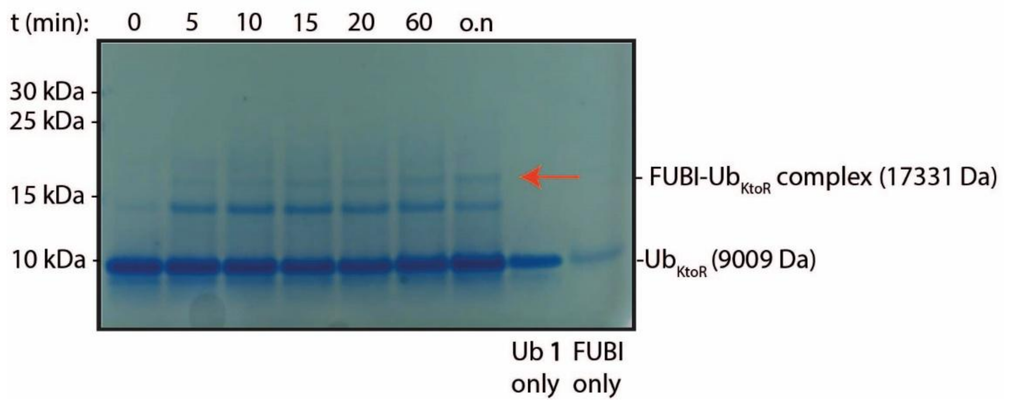

Figure S19. FUBI-Ub$_{KtoR}$ conjugate formed via selective lysine acylation at pH 10.5 at the red arrow. Fluorescent scan of SDS-Page analysis (upper gel) of acylation of FUBI with activated Ub (**1**) followed over the indicated time period ($\lambda_{ex/em}$ = 473/530 nm), and Coomassie Blue Stain (lower panel).

Proteomics mass spectrometry measurements

Samples were loaded onto a 4-12 % Bis-Tris gradient gel (Invitrogen) and subsequently the appropriate band was cut out. Gel slices were first washed 3x with water, and subsequently subjected to reduction with 10 mM dithiothreitol, alkylation with 50 mM of iodoacetamide, and in-gel endo-Glu and trypsin digestion using a Proteineer DP digestion robot (Bruker). After addition of the digestive enzymes and swelling of the bands, the bands were transferred to Eppendorf vials and the bands were covered in 25 mM NH_4HCO_3 pH 8.3 overnight at 37 °C and the peptides were extracted from the gel slices with with 50/50/0.1 v/v/v water/acetonitril/formic acid. Finally peptides were lyophilized.

Tryptic peptides were dissolved in water/formic acid (100/0.1 v/v) and subsequently analyzed by on-line C18 nanoHPLC MS/MS with a system consisting of an Ultimate3000nano gradient HPLC system (Thermo, Bremen, Germany), and an Exploris480 mass spectrometer (Thermo). Fractions were injected onto a cartridge precolumn (300 μm × 5 mm, C18 PepMap, 5 um, 100 A, and eluted via a homemade analytical nano-HPLC column (50 cm × 75 μm; Reprosil-Pur C18-AQ 1.9 um, 120 A (Dr. Maisch, Ammerbuch, Germany). The gradient was run from 2% to 40 % solvent B (20/80/0.1 water/acetonitrile/formic acid (FA) v/v) in 30 min. The nano-HPLC column was drawn to a tip of ∼10 μm and acted as the electrospray needle of the MS source. The mass spectrometer was operated in data-dependent MS/MS mode for a cycle time of 3 seconds, with a HCD collision energy at 30 V and recording of the MS2 spectrum in the orbitrap, with a quadrupole isolation width of 1.2 Da. In the master scan (MS1) the resolution was 120,000, the scan range 400-1500, at standard AGC target @maximum fill time of 50 ms. A lock mass correction on the background ion m/z = 445.12 Da was used. Precursors were dynamically excluded after n=1 with an exclusion duration of 10 s, and with a precursor range of 20 ppm. Charge states 2-5 were included. For MS2 the first mass was set to 110 Da, and the MS2 scan resolution was 30,000 at an AGC target of 100% at maximum fill time of 60 ms.

In a post-analysis process, raw data were first converted to peak lists using Proteome Discoverer version 2.2 (Thermo Electron), and submitted to the combined Uniprot database (Homo sapiens, 20596 entries, using Mascot v. 2.2.07 (www.matrixscience.com) for protein identification. Mascot searches were with 10 ppm and 0.02 Da deviation for precursor and fragment mass, respectively, and endo-Glu and trypsin as enzyme. Up to two missed cleavages were allowed. Methionine oxidation and acetyl on protein N-terminus were set as a variable modification; carbamidomethyl on Cys, were set as a fixed modification. Protein FDR was set to 1 %. Normalization was on total peptide amount.

References

[1] J. H. Mikkelsen, M. B. F. Gustafsson, T. Skrydstrup, K. B. Jensen, *Bioconjug. Chem.* **2022**, *33*, 625–633.
